# Pretreatment of metanephric mesenchymal cells with catalpol mitigates acute kidney injury through VEGF-A secretion via multiple mechanisms

**DOI:** 10.1186/s13287-026-04914-9

**Published:** 2026-03-28

**Authors:** Pengcheng Ji, Yuansheng Xie, Wenkai Guo, Quanhang Jiang, Jingru Bi, Bing Han, Zhiwei Yin, Bo Fu

**Affiliations:** 1 Medical School of Chinese PLA, Beijing, 100853 China; 2 Department of Nephrology, First Medical Center of Chinese PLA General Hospital, State Key Laboratory of Kidney Diseases, National Clinical Research Center for Kidney Diseases, Beijing Key Laboratory of Medical Devices and Integrated Traditional Chinese and Western Drug Development for Severe Kidney Diseases, Beijing Key Laboratory of Digital Intelligent TCM for the Prevention and Treatment of Pan-vascular Diseases, Key Disciplines of National Administration of Traditional Chinese Medicine (zyyzdxk-2023310), Beijing, 100853 China; 3 School of Medicine, Nankai University, Tianjin, 300071 China; 4 Department of Nephrology, The Second Hospital of Jilin University, Jilin, 130062 China; 5 Department of Nephrology, Tianjin Union Medical Center, The First Affiliated Hospital of Nankai University, Tianjin, 300121 China; 6 College of Integrated Chinese and Western Medicine, Hebei Medical University, Shijiazhuang, 050017 China

**Keywords:** Catalpol, Metanephric mesenchymal cells, Acute kidney injury, VEGF-A, Necroptosis

## Abstract

**Background:**

Metanephric mesenchymal cells (MMCs) hold therapeutic potential for acute kidney injury (AKI), but their efficacy is limited, and the mechanisms underlying their action remain unclear. This study aimed to investigate whether catalpol-pretreated MMCs (MMCs-cata) could enhance the efficacy of AKI treatment by regulating key signaling pathways.

**Methods:**

An AKI model was established in C57BL6 mice via intraperitoneal injection of cisplatin, and the therapeutic effects of MMCs-cata were compared with those of untreated MMCs. RNA-Seq was performed to analyze differentially expressed genes, Western blotting and ELISA were used to measure VEGF-A levels in MMC-cata and the supernatants. An in vitro model of cisplatin-induced renal tubular epithelial cell injury was developed to investigate the signaling pathways upstream and downstream of VEGF-A. The role of VEGF-A/VEGFR2 was confirmed through experiments involving gene silencing, neutralizing antibodies, and VEGFR2 blockade. Additionally, molecular docking simulations and Western blotting were performed to explore the effects of catalpol on the Wnt signaling pathway.

**Results:**

MMCs-cata significantly improved renal function in AKI model mice by suppressing inflammation, oxidative stress, and necroptosis. RNA-Seq, Western blotting and ELISA revealed the activation of VEGF-related genes in MMCs-cata along with elevated intracellular and supernatant VEGF-A levels. In both the in vivo and in vitro models, silencing VEGF-A in MMCs-cata, neutralizing VEGF-A in the supernatant, or blocking VEGFR2 in tubular epithelial cells abolished the protective effects of MMCs-cata, while exogenous VEGF-A supplementation alone exerted protective effects. Mechanistic studies indicated that MMCs-cata activated the p38 pathway and suppressed the STAT3 pathway. Molecular docking and Western blotting confirmed that catalpol binds to Wnt3A, activating the canonical Wnt pathway to drive VEGF-A secretion.

**Conclusion:**

Catalpol pretreatment enhances the therapeutic efficacy of MMCs by activating the canonical Wnt pathway to promote VEGF-A secretion. VEGF-A interacts with VEGFR2 on renal tubular epithelial cells, likely through both p38 pathway activation and STAT3 pathway inhibition, thereby suppressing inflammation, oxidative stress, and necroptosis to alleviate AKI. This study provides novel insights into the integration of traditional Chinese medicine components with stem cell therapy for AKI management.

**Supplementary Information:**

The online version contains supplementary material available at 10.1186/s13287-026-04914-9.

## Background

Acute kidney injury (AKI) is a common and significant clinical issue [1]. Drug-induced kidney injury, including that caused by cisplatin, is a major cause of AKI [2–4]. Currently, no specific treatments have emerged to mitigate acute kidney injury or accelerate recovery [5]. Therefore, it is important to search for new therapies for AKI that are safe and effective.

Metanephric mesenchymal cells (MMCs) originate from the metanephros during embryonic kidney development and can differentiate into various types of intrinsic kidney cells [6, 7]. Currently, reports in the literature indicate that MMCs have therapeutic effects on acute kidney injury (AKI) [8, 9], but the mechanisms by which MMCs treat AKI remain unclear, and their efficacy needs further improvement. Some studies suggest that pretreating transplanted cells with small-molecule drugs to enhance their bioactivity and improve their growth microenvironment may be an effective strategy [10], to promote tissue repair. Additionally, small-molecule compound pretreatment of cells offers advantages in terms of convenience and safety [11, 12].

Catalpol, a small-molecule drug, is a major active component of Rehmannia glutinosa (Di Huang), a traditional kidney-tonifying medicine, has been shown to have various biological effects [13, 14]. Catalpol is widely used to treat diseases such as diabetes and Alzheimer’s disease, with mechanisms potentially related to reducing oxidative stress and anti-inflammatory and antiapoptotic effects [15, 16]. However, whether pretreatment with catalpol can enhance the efficacy of MMCs against AKI and the underlying mechanisms by which it enhances efficacy and exerts therapeutic effects remain unclear.

In this study, we utilized catalpol-pretreated MMCs and discovered that catalpol enhanced the renal repair effects of MMCs in AKI. Catalpol activates the canonical Wnt pathway by binding to Wnt3A in MMCs and promotes the secretion of VEGF-A by MMCs, thereby alleviating kidney injury by reducing inflammation, oxidative stress, and necroptosis in renal tubular epithelial cells. This pretreatment strategy enhanced the paracrine function and cellular activity of MMCs, thereby enhancing the ability of MMCs to repair the kidneys in AKI.

## Methods

### Animal model and cell injection

The work is reported in accordance with the ARRIVE guidelines 2.0. In this study, 8-week-old male C57BL6 mice were selected as the model and were purchased from the Experimental Animal Center of the PLA General Hospital. The mice were given free access to food and water and housed on a 12-hour light/dark cycle. All animal experiments were performed under ethical supervision and approved by the Institutional Animal Care and Use Committee of the Chinese PLA General Hospital (No. 2024-X20-97). The mice were randomly assigned to different treatment groups, with 6 mice in each group. All experimental animals were individually marked with ear tags and assigned to groups through a computerized randomization process. This procedure involved selecting an arbitrary starting point in a random number table, sequentially generating N values along a fixed direction, and calculating group allocation using modulo division with the total group count as the divisor. The subjects were assigned to groups on the basis of the resulting remainders, where a zero remainder placed the animal in the highest numbered group. Prior to modeling, all groups of mice were fasted for 24 h.

The cisplatin-induced AKI model is a relatively well-established system, with 20 mg/kg cisplatin being a commonly used dose in mouse models [17–19]. To increase the severity of injury and achieve more pronounced tubular basement membrane exposure—enabling better observation of MMC migration and binding to damaged sites—we used 25 mg/kg cisplatin in this study, by intraperitoneal injection of a 1 mg/ml cisplatin solution in PBS in cisplatin-induced AKI group (CP group). The control group (Ctrl group) received an equivalent volume of PBS. To avoid the influence of the cisplatin solution on MMCs, MMCs were injected intraperitoneally 3 h after cisplatin injection. To explore the efficacy of intraperitoneal injection versus tail vein injection of MMC in cisplatin-induced acute kidney injury, after cisplatin intraperitoneal injection for 3 h, the intraperitoneal MMC treatment group (MMC-abdominal group) mice were treated with 1 × 10^6 MMC in 1 ml PBS by intraperitoneal injection, and the tail vein MMC treatment group (MMC-tail vein group) mice were treated with 1 × 10^6 MMC in 1 ml PBS by tail vein injection. The MMC-con group received untreated MMCs (2 × 10^6 in 0.5 ml of PBS per mouse), while the MMC-cata group received MMCs pretreated with 1 µM catalpol for 24 h (2 × 10^6 in 0.5 ml of PBS per mouse). The CP group and the Ctrl group were injected with 0.5 ml of PBS solution.

Seventy-two hours after cisplatin or PBS injection, the mice were euthanized by cervical dislocation, and whole blood and kidney tissues were collected. Blood samples were allowed to stand at room temperature for 1 h and then centrifuged at 3000 rpm for 10 min at 4 °C to separate the serum. Blood creatinine and urea nitrogen levels were measured using a small animal automatic biochemical analyzer (Hitachi 7150). The assignment process was conducted independently without experimenter involvement, with researchers receiving only the final group allocations. To ensure objectivity, outcome assessment and statistical analysis were performed by independent evaluators, with a minimum of two investigators conducting parallel verification of all the analytical results.

To monitor the intraperitoneal distribution of fluorescently labeled MMCs, real-time imaging was performed using an IVIS Lumina system at designated time points postinjection. Anesthesia was maintained by inhalation of 1% isoflurane throughout the imaging procedure to ensure optimal immobilization.

### Measurement of tissue MDA, GSH, and T-SOD levels

The levels of MDA, GSH, and T-SOD in tissues were measured with an MDA detection kit (G4302; ServiceBio, China), a GSH detection kit (G4305; ServiceBio, China), and a T-SOD detection kit (S0101; Beyotime, China) according to the manufacturer’s instructions.

### Histological assay and scoring

After fixation in 4% paraformaldehyde, kidney tissues were embedded in paraffin and sectioned to a thickness of 3–5 μm. The histopathological sections were subjected to periodic acid–Schiff (PAS) staining, and renal tubular pathological damage was observed and scored under a light microscope. The scoring criteria for tubular necrosis included tubular dilation, brush border loss, cast formation, and the degree of tubular necrosis. Ten randomly selected nonoverlapping fields were scored on the basis of the area of damage, with the following standards: 0, none; 1, ≤ 25%; 2, 26–50%; 3, 51–75%; and 4, ≥ 76%.

The immunohistochemical staining procedures were conducted as previously described [20]. The antibodies used, which were diluted as indicated, included those against MLKL (1:100; GB115699; ServiceBio, China) and VEGFR2 (1:100; GB11190; ServiceBio, China).

### Bioluminescence imaging

To monitor the localization of the labeled metanephric mesenchymal cell mCherry signal in mice in real time, bioluminescence imaging was performed using an IVIS Lumina system. mCherry signals were captured at different time points before and after cell injection.

### Cell culture and treatment

Metanephric mesenchymal stem cells (MMCs) were obtained from our research group by extraction methods previously described in the literature [7]. TCMK-1 cells were purchased from the Cell Bank of the Chinese Academy of Sciences, and those from passages 4–6 were used for the experiments. Mesenchymal stem cells were cultured in α-MEM supplemented with 10% fetal bovine serum (FBS) and 1% penicillin‒streptomycin, while TCMK-1 cells were cultured in DMEM/F12 supplemented with 5% FBS and 1% penicillin‒streptomycin. All the cultures were maintained in a 37 °C incubator with 5% CO2, and the culture medium was changed every two days.

MMCs were pretreated as follows. First, 24 h prior to cell injection into the mouse peritoneal cavity, when cell confluence reached approximately 80%, the culture medium was changed. In the control MMC (MMC-con) group, the medium was changed to regular α-MEM, while in the catalpol-pretreated MMC (MMC-cata) group, the medium was changed to α-MEM containing 1 µM catalpol supplemented with 10% FBS. The cells were then cultured for an additional 24 h, followed by digestion with 0.5% EDTA-trypsin, resuspension in PBS, and preparation for injection. Prior to injection, the catalpol in the culture medium was removed by washing with PBS; thus, no catalpol was present in the cell suspensions of either group.

The preparation method for the MMC-conditioned medium was as follows. After the cells were seeded, the culture medium was replaced when the cell confluence reached approximately 80%. In the con-CM group, the medium was changed to regular α-MEM, while in the cata-CM group, the medium was changed to α-MEM supplemented with 1 µM catalpol and 10% FBS. The MMCs were cultured for 24 h until they reached 100% confluence. The supernatant was discarded, and the cells were washed twice with PBS and then incubated in serum-free DMEM/F12 for 48 h. The supernatant was then collected, centrifuged at 1200 rpm for 5 min, filtered through a 0.22 μm filter, and stored at −80 °C. One day before the experiments were performed, the supernatant was thawed at 4 °C in a refrigerator, and then the MMC-conditioned medium was mixed with the regular culture medium at a 1:1 ratio, after which the serum concentration was adjusted to ensure consistency across groups.

To observe the fate and migration of MMCs after they entered the mice, we constructed MMCs with spontaneous mCherry fluorescence using lentiviral transfection and puromycin selection, allowing observation using a small animal bioluminescence imaging system. The lentivirus was purchased from Suzhou Jima Biological Company (Jiangsu, China).

The negative control siRNA and VEGF-A siRNA were purchased from Suzhou Jima Biological Company (Jiangsu, China) for the experiments involving siRNA-mediated knockdown of vascular endothelial growth factor-A (VEGF-A). Cell transfection was performed using Lipofectamine 3000 reagent (Invitrogen, USA) according to the manufacturer’s instructions. The sequences of the siRNAs used are provided in supplemental materials Table S2.

In the in vitro experiments involving the blockade of VEGFR2, the application of VEGF-A neutralizing antibodies, and VEGF-A treatment, the concentrations of various small-molecule drugs added were determined according to the manufacturer’s instructions, previous literature reports, and preliminary experimental results [21–23]. Ultimately, 5 ng/ml VEGF-A (HY-P72280, MCE, USA), 0.1 µg/ml VEGF-A neutralizing antibody (sc-7249, Santa Cruz, USA), 10 nM VEGFR2 blocker Ki8751 (HY-12038, MCE, USA), and 5 µM VEGFR3 blocker MAZ51 (HY-116624, MCE, USA) were used.

CCK-8 and LDH assays were performed to assess cell viability and injury. A CCK-8 assay kit (CK-04, Dojindo, Japan) was used according to the manufacturer’s instructions to test cell viability, and the absorbance of the samples was measured at 450 nm using a UV spectrophotometer. An LDH assay kit (C0018; Beyotime, China) was used according to the manufacturer’s instructions to assess the degree of cell damage, with the absorbance of the samples measured at 450 nm using a UV spectrophotometer.

To evaluate oxidative stress, ROS and SOD levels were measured. The presence of ROS in TCMK-1 cells from different treatment groups was evaluated using a ROS fluorescent probe (S0033; Beyotime, China) following the provided protocol. FITC (green fluorescence) was observed and photographed under a fluorescence microscope. For quantitative measurements, the cells were washed twice with PBS, digested in a 1.5 ml EP tube, and incubated with the fluorescent probe at 37 °C for 30 min. Measurements were taken using a microplate reader (Tecan, Switzerland) with an excitation wavelength of 488 nm and an emission wavelength of 525 nm. The total SOD level in the cells was measured using a T-SOD detection kit (S0101; Beyotime, China) following the manufacturer’s instructions. Measurements were taken at 450 nm with a microplate reader.

The VEGF-A content in the conditioned medium of mesenchymal stem cells from different treatment groups was detected using an ELISA kit (SEKP-0030; Solarbio, China) according to the provided protocol.

The method for cell immunofluorescence detection was as previously reported in the literature [7]. The reagents used included an anti-VEGFR2 antibody (1:100; 26415-1-AP; Proteintech, China) and DAPI (Abcam, USA).

The sample preparation steps for the cell RNA sequencing experiment were reported previously [24]. The RNA libraries were sequenced on the Illumina NovaSeq™ 6000 platform, and the data were analyzed by LC Bio Technology Co., Ltd. (Hangzhou, China).

### Western blot analysis

Following the collection of kidney and cellular samples from the animals, protein lysates were prepared by lysing the samples in RIPA lysis buffer (Cell Signaling Technology, USA) on ice for 30 min. The lysates were then centrifuged at 12,000 rpm for 30 min at 4 °C. Protein concentrations were determined using BCA working solution (Life Technologies, USA) according to the manufacturer’s instructions. SDS‒PAGE protein separation and subsequent Western blot analysis were conducted following standard protocols. Imaging was performed using a ChemiDoc-It imaging system (UVP, USA). The following antibodies were utilized in the experiments: anti-RIP, anti-RIP3, anti-MLKL, and their phosphorylated forms (1:1000, 98110T, CST, USA); anti-GAPDH (1:10000, 60004-1-Ig, Proteintech, China); anti-β-catenin (1:5000, 51067-2-AP, Proteintech, China); anti-TCF-4 (1:5000, 22337-1-AP, Proteintech, China); anti-p-p65 (1:1000, 82335-1-RR, Proteintech, China); anti-TNF-α (1:1000, 80258-6-RR, Proteintech, China); anti-Wnt3A (1:2000, GB113750, ServiceBio, China); anti-KIM-1 (1:1000, ab316854, Abcam, USA); anti-VEGF-A (1:500, sc-57496, Santa Cruz Biotechnology, USA); anti-p38 (1:1000, 9212, CST, USA); anti-p-p38 (1:1000, 4511, CST, USA); anti-STAT3 (1:1000, 80149-1-RR, Proteintech, China); anti-p-STAT3 (1:1000, 28945-1-AP, Proteintech, China) and an anti-HRP-conjugated secondary antibody (1:1000, Beyotime, China).

### Molecular docking and molecular dynamics simulation

Molecular docking was performed using the Site-Find module of MOE (Chemical Computing Group Inc., Canada) to identify binding sites for small molecules. Proteins and small molecules underwent hydrogen addition and physiological pH adjustments prior to docking, which was executed using the MOE-DOCK module. The London ΔG scoring function was employed to calculate the docking scores, and 300 docking poses were retained. The GBVI/WAS ΔG scoring function was subsequently used for precise docking, ultimately preserving the conformation with the best score for each molecule, along with the top 10 ranked conformations.

Molecular dynamics simulations were conducted using Amber software (University of California, San Francisco, USA). The Amber traj program was utilized to process the simulation trajectories, and the conformations of the simulated system were output at intervals of 2 fs. For the complex systems, the RMSDs were calculated for the protein αC atoms, receptor backbone, and small molecules. The output conformations were visualized using Grace software.

### Surface plasmon resonance

SPR was employed to characterize the binding kinetics, thermodynamic parameters, and equilibrium constants of the molecular interactions, enabling precise evaluation of the binding affinity between the Wnt3A protein and small-molecule ligands. The experimental procedures were conducted following established protocols [25] with minor modifications. Briefly, SPR measurements were performed on a Biacore 8 K system. Recombinant wild-type Wnt3A was diluted to 50 µg mL − 1 in 10 mM acetate buffer (pH 4.5) and immobilized onto a CM5 sensor chip via amine coupling at a constant flow rate of 10 µL min − 1 for 600 s. Real-time binding interactions were monitored using the Biacore 8 K platform. Additionally, the specific binding between catalpol and Wnt3A was examined under identical conditions.

### Statistical analysis

The data are presented as the means ± standard deviations. A t test was used to analyze differences between two groups, while one-way ANOVA was used for multiple group comparisons. Statistical analyses were conducted using GraphPad Prism version 8.2.1 (GraphPad Software, San Diego, CA, USA), with a significance threshold set at a two-sided p value of < 0.05.

## Results

### Pretreatment of metanephric mesenchymal cells with catalpol more effectively alleviates cisplatin-induced acute kidney injury

**Fig. 1:**
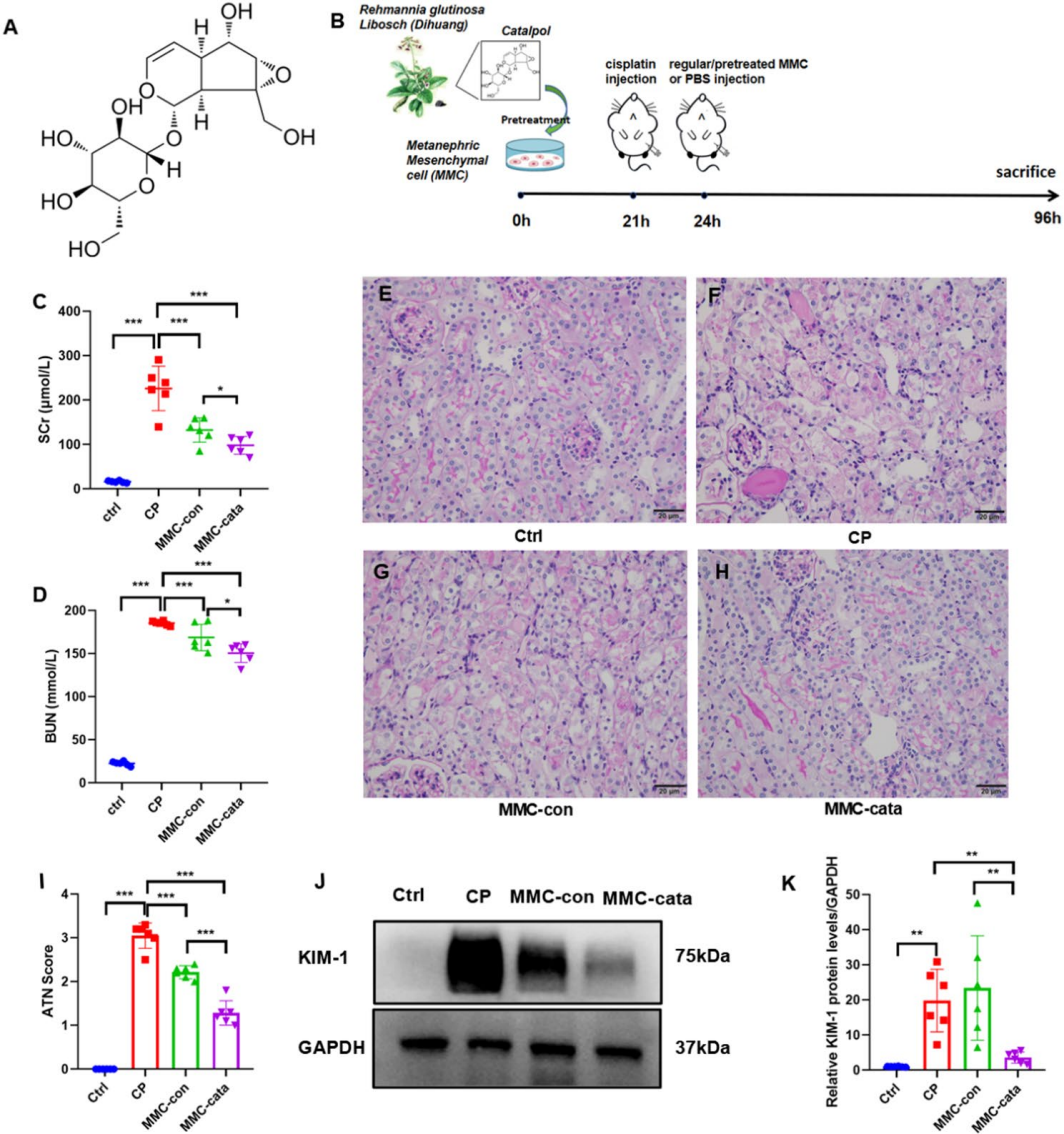
Catalpol pretreatment enhances the protective effect of MMC on cisplatin-induced acute kidney injury. (**A**) Molecular structure of catalpol. (**B**) Flowchart of the experimental design. (**C**, **D**) SCr and BUN levels (*n* = 6). (**E**-**I**) PAS staining of kidney tissue Sect. (400×) and semiquantitative analysis of histological scores (*n* = 6). (J-K) Western blot images and quantitative analysis of KIM-1 expression in kidney tissues (*n* = 6). *Ctrl* control group, *CP* cisplatin model group, *MMC-con* normal MMC treatment group, *MMC-cata* catalpol-pretreated MMC treatment group. ***P < 0.001, **P < 0.01, *P < 0.05

We established a cisplatin-induced acute kidney injury (AKI) model to evaluate the protective effects of catalpol-pretreated metanephric mesenchymal cells (MMCs) on AKI. We previously found that intraperitoneal injection is more effective than tail vein injection (Supplementary Fig S2); therefore, this experiment used intraperitoneal injection to treat the mice.We found that mice injected with MMCs following cisplatin administration had lower SCr and BUN levels than those injected with PBS did, with the MMC-cata group showing significantly lower SCr and BUN levels than the MMC-con group (Fig. 1C, D). Periodic acid–Schiff (PAS) staining was used to assess histopathological changes. Renal tubular injury was quantified using the acute tubular necrosis (ATN) score. In the cisplatin-induced AKI model, characteristic renal injuries included tubular necrosis, tubular dilation, cast formation in the lumen, and loss of the brush border. Pathological scoring revealed that the ATN scores of the MMC-cata and MMC-con groups were significantly lower than those of the CP group, with the former showing the greatest reduction (Fig. 1E–I). Western blot analysis revealed that the expression of KIM-1 in the MMC-cata group was significantly lower than that in the MMC-con group (Fig. 1J, K). These findings suggest that MMCs pretreated with catalpol more effectively alleviate cisplatin-induced AKI.

### Catalpol pretreated MMCs alleviate cisplatin-induced acute kidney injury by reducing inflammation, oxidative stress, and necroptosis

**Fig. 2:**
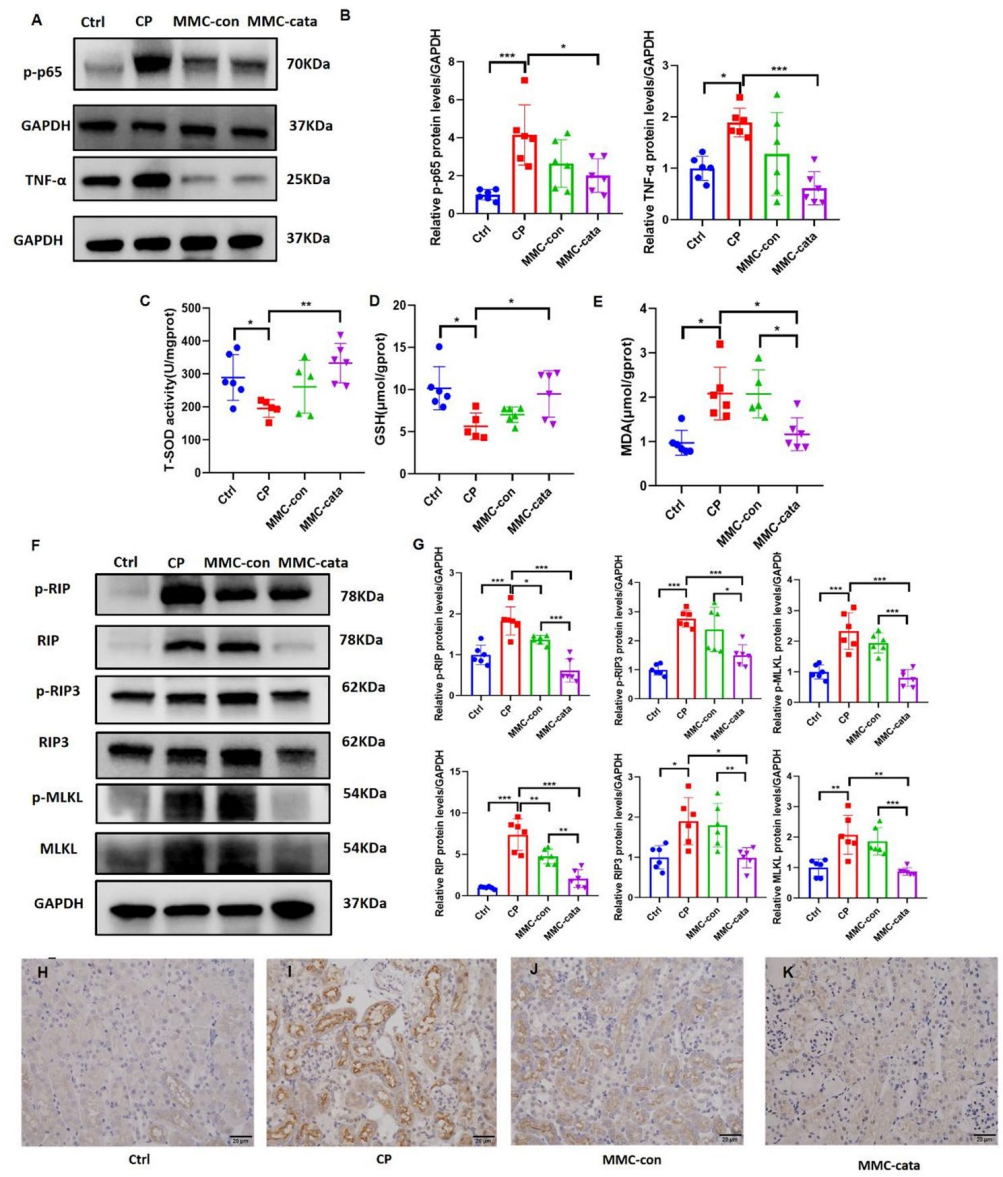
Catalpol-pretreated MMCs reduce cisplatin-induced acute kidney injury by alleviating inflammation, oxidative stress, and necroptosis. (**A**, **B**) Western blot analysis of the expression of the inflammatory markers p-p65 and TNF-α in the kidneys of mice from different groups and statistical analysis (*n* = 6). (**C**–**E**) Expression levels of the oxidative stress indicators T-SOD, GSH, and MDA in kidney tissue (*n* = 6). (**F**, **G**) Western blot analysis of the expression of the necroptosis markers RIP, RIP3, and MLKL and their phosphorylated forms in the kidneys of mice from different groups and statistical analysis (*n* = 6). (**H**–**K**) Immunohistochemical staining images showing the expression of the necroptosis marker MLKL in kidney tissues from different groups of mice (400× magnification). *Ctrl* control group; *CP* cisplatin model group; *MMC-con* normal MMC treatment group; *MMC-cata* catalpol-pretreated MMC treatment group. ***P < 0.001, **P < 0.01, *P < 0.05

In mice injected with cisplatin, intraperitoneal administration of catalpol-pretreated MMCs resulted in reduced inflammation, oxidative stress, and necroptosis. Western blot analysis revealed that the expression levels of the inflammatory markers p-p65 and TNF-α in the MMC-cata group were significantly lower than those in the CP group and the MMC-con group (Fig. 2A, B). Additionally, compared with those in the CP group, the levels of GSH and T-SOD in the MMC-cata group were significantly greater (Fig. 2C, D), while the MDA level in the MMC-cata group was significantly lower than that in the CP group and the MMC-con group (Fig. 2E). The expression levels of the necroptosis markers RIP, RIP3, and MLKL, as well as their phosphorylated protein forms, were significantly lower in the MMC-cata group than in the CP group and the MMC-con group (Fig. 2F, G). Immunohistochemical staining revealed that the expression of the necroptosis marker MLKL in proximal tubular epithelial cells was lower in the MMC-cata treatment group than in the MMC-con group (Fig. 2H–K). These findings suggest that the therapeutic efficacy of catalpol-pretreated MMCs against AKI is enhanced through the mitigation of inflammation, oxidative stress, and necroptosis through multiple pathways.

### Conditioned medium from MMC-cata alleviates cisplatin-induced injury by reducing inflammation, oxidative stress, and necroptosis in vitro

**Fig. 3:**
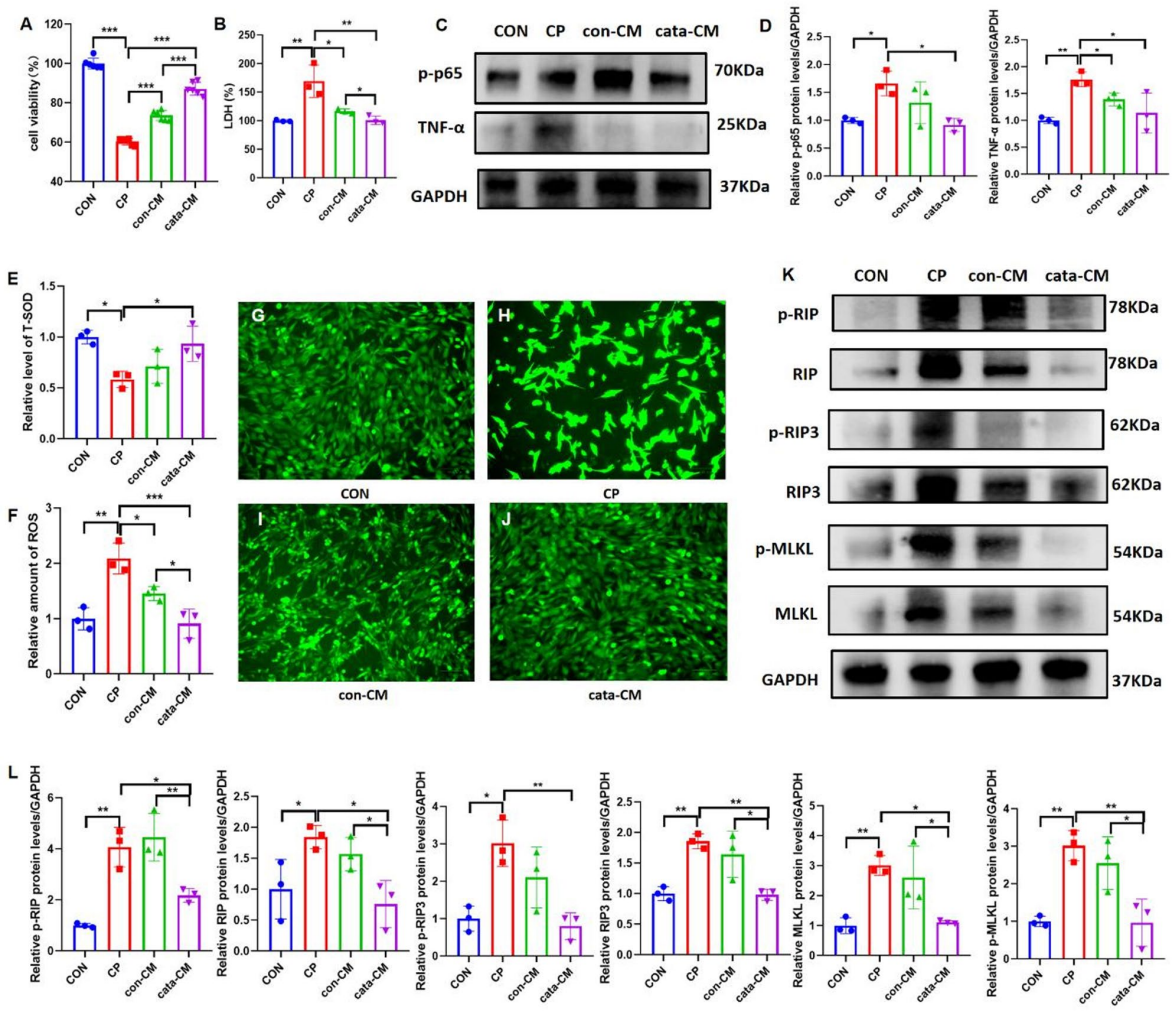
MMC-conditioned medium reduces cisplatin-induced injury by alleviating inflammation, oxidative stress, and necroptosis in vitro. (**A**, **B**) CCK-8 and LDH assay results showing the proliferative activity and cytotoxicity of TCMK-1 cells in different groups. (**C**, **D**) Western blot images and statistical analysis (*n* = 3) showing the expression levels of the inflammatory markers p-p65 and TNF-α in TCMK-1 cells across different groups. (**E**-**J**) Oxidative stress indicators, including T-SOD levels, ROS levels, and ROS staining intensity, in TCMK-1 cells (*n* = 3). (**K**, **L**) Western blot images and statistical analysis (*n* = 3) showing the expression levels of the necroptosis markers RIP, RIP3, and MLKL, as well as their phosphorylated forms, in TCMK-1 cells across different groups. *CON* control group; *CP* cisplatin injury group; *con-CM* conventional MMC supernatant group; *cata-CM* catalpol-pretreated MMC supernatant group. ***P < 0.001, **P < 0.01, *P < 0.05

To investigate whether MMCs secrete certain effective components that contribute to injury alleviation, conditioned medium from MMCs was used to treat cisplatin-injured TCMK-1 cells under in vitro conditions. The in vitro experiments demonstrated that compared with control cells, cisplatin-injured TCMK-1 cells treated with either conventional MMC-conditioned medium (con-CM) or catalpol-pretreated MMC-conditioned medium (cata-CM) exhibited increased cell viability and reduced intracellular LDH levels (Fig. 3A, B). The expression levels of the inflammatory markers p-p65 and TNF-α in the cata-CM group were significantly lower than those in the CP group (Fig. 3C, D). Compared with the CP group, the cata-CM group presented increased T-SOD activity (Fig. 3E), reduced ROS levels (Fig. 3F–J), and decreased expression levels of the necroptosis markers RIP, RIP3, and MLKL, as well as their phosphorylated forms (Fig. 3K, L). While the effects on inflammation and oxidative stress levels did not significantly differ between the cata-CM and con-CM groups, the cata-CM group demonstrated superior therapeutic efficacy in mitigating necroptosis. These findings suggest that the application of catalpol-pretreated MMC-conditioned medium alone can alleviate cisplatin-induced TCMK-1 cell injury by reducing inflammation, oxidative stress, and necroptosis.

### Catalpol pretreatment promotes VEGF-A secretion by MMCs

**Fig. 4:**
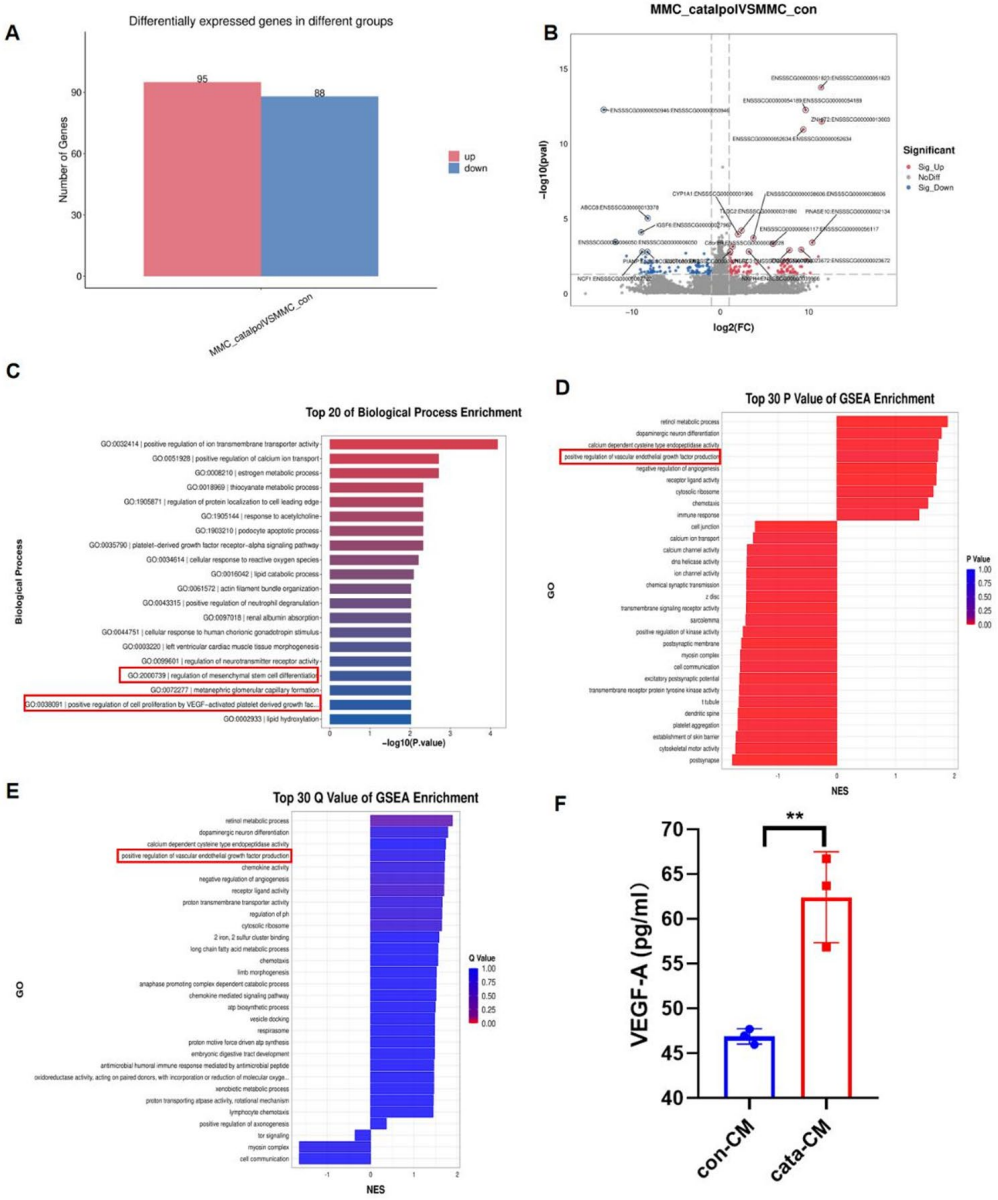
The enhanced efficacy of MMCs following catalpol pretreatment may be associated with VEGF-A secretion. (**A**) A total of 183 differentially expressed genes were identified between the ordinary MMC group and the catalpol-pretreated MMC group. (**B**) RNA-seq volcano plot illustrating the genes that were differentially expressed between the cisplatin treatment group and ordinary TCMK-1 cells (*n* = 4), with red indicating upregulated gsenes, blue representing downregulated genes, and gray denoting nondifferentially expressed genes. (**C**) GO enrichment analysis revealed the top 20 biological processes associated with the identified pathways. (**D**,** E**) Results from the GSEA of the GO terms ranked by P value and Q value, indicating the activation of retinol metabolism and VEGF production-related pathways in catalpol-pretreated MMCs. (**F**) ELISA results demonstrating that the content of VEGF-A in the conditioned medium from the catalpol-pretreated MMC group was significantly greater than that in the control group (*n* = 3). con-CM: conventional MMC supernatant group; cata-CM: catalpol-pretreated MMC supernatant group. **P < 0.01

To further elucidate the mechanisms by which MMCs exert their therapeutic effects, we conducted RNA sequencing on MMCs under normal conditions and following catalpol treatment. A total of 183 genes were differentially expressed between the conventional MMC and catalpol-pretreated MMC groups, including 95 upregulated genes and 88 downregulated genes (Fig. 4A). Gene Ontology (GO) analysis revealed that genes related to mesenchymal stem cell differentiation and pathways associated with VEGF-mediated platelet-derived growth factor-induced proliferation were activated in catalpol-pretreated MMCs (Fig. 4C). Gene set enrichment analysis (GSEA) of the GO terms revealed that pathways related to VEGF synthesis were activated in catalpol-pretreated MMCs (Fig. 4D, E). ELISAs of conditioned medium from control MMCs and catalpol-pretreated MMCs demonstrated that the level of VEGF-A was significantly greater in the catalpol-pretreated group than in the control group (Fig. 4F). These findings suggest that catalpol pretreatment promotes VEGF-A secretion by MMCs. Combined with our in vivo imaging results showing that MMCs primarily remained in situ after intraperitoneal injection (Supplementary Fig S3) and the observation that conditioned medium from MMCs alone alleviated cisplatin-induced injury in renal tubular epithelial cells, we speculate that the enhanced therapeutic efficacy of catalpol-pretreated MMCs may be related to VEGF-A secretion.

### Knockdown of VEGF-A in MMCs abrogates the protective effect of catalpol-pretreated MMCs against AKI

**Fig. 5:**
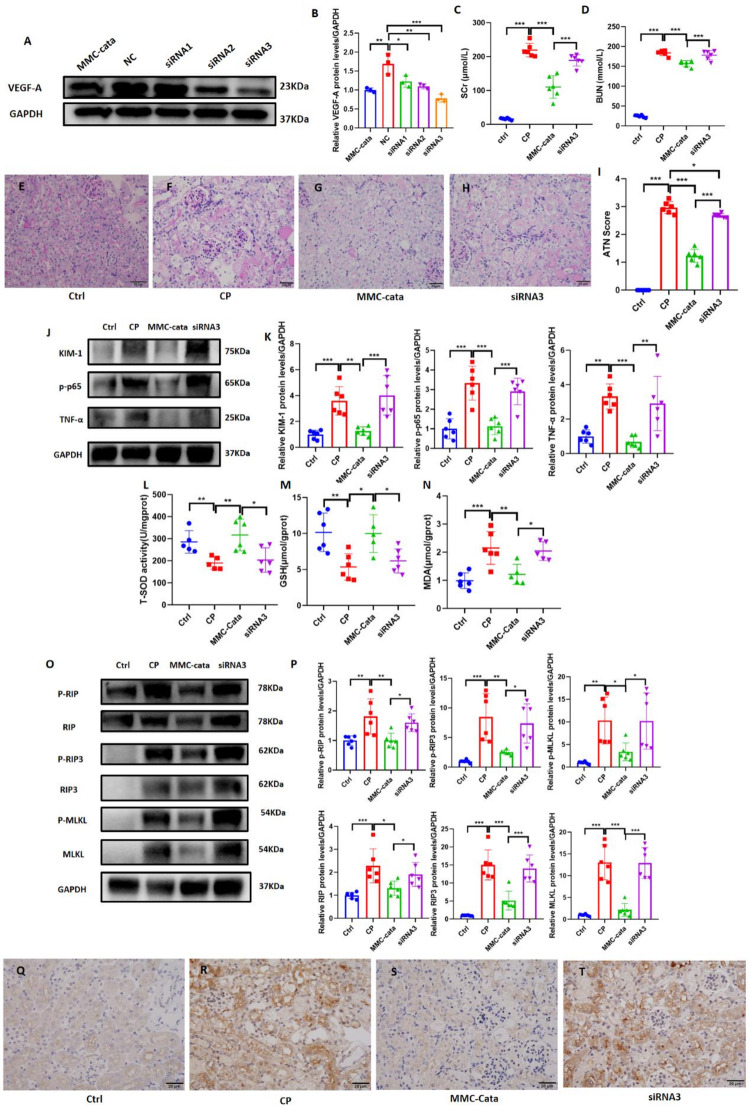
Knockdown of VEGF-A in MMCs Abrogates the Protective Effect of Catalpol-Pretreated MMCs against AKI. (**A**, **B**) Western blot showing the expression of VEGF-A in MMCs transfected with different siRNAs and treated with catalpol for 24 h (*n* = 3). (**C**, **D**) Serum levels of SCr and BUN in the different groups of mice (*n* = 6). (**E**-**I**) Semiquantitative analysis of histopathological scores (*n* = 6) and representative images of PAS-stained kidney tissue sections from different groups of mice (400×). (**J**, **K**) Western blot showing the expression level of the injury marker KIM-1 and the inflammatory markers p-p65 and TNF-α in the kidneys of mice from different groups and statistical analysis (*n* = 6). (L–N) Levels of the oxidative stress markers T-SOD, GSH, and MDA in the kidney tissues of mice from different groups (*n* = 6). (**O**, **P**) Western blot showing the expression of the necroptosis markers RIP, RIP3, and MLKL, as well as their phosphorylated forms, in the kidneys of mice from different groups and statistical analysis (*n* = 6). (**Q**-**T**) Immunohistochemical staining images showing the expression of the necroptosis marker MLKL (400×). Groups in panels A-B: MMC-cata: catalpol-pretreated MMCs; NC: MMCs transfected with the empty vector; siRNA1: MMCs transfected with siRNA1; siRNA2: MMCs transfected with siRNA2; siRNA3: MMCs transfected with siRNA3. Groups in panels C-T: Ctrl: Control group; CP: cisplatin model group; MMC-cata: catalpol-pretreated MMC treatment group; siRNA3: siRNA3-transfected MMC treatment group. ***P < 0.001, **P < 0.01, *P < 0.05

To validate the role of VEGF-A in the enhanced therapeutic efficacy of catalpol-pretreated MMCs, we selected three different siRNAs targeting distinct sites of the VEGF-A gene for interference. Western blotting indicated that siRNA3, which targets the 470 site, exhibited the most effective knockdown (Fig. 5A, B). After the MMCs were transfected with empty plasmids or siRNA3 plasmids, the cells were pretreated with catalpol for 24 h and then injected into mice with cisplatin-induced injury. Compared with mice injected with siRNA3-treated MMCs and the mice in the CP group, mice injected with MMCs-cata presented significantly lower SCr and BUN levels (Fig. 5C, D). Periodic acid–Schiff (PAS) staining was used to assess histopathological changes, and renal tubular injury was quantified using the acute tubular necrosis (ATN) score. Pathological scoring revealed that compared with the CP and siRNA3 groups, the MMC-cata group had significantly lower ATN scores (Fig. 5E–I). Western blot analysis revealed that the expression levels of KIM-1 and the inflammatory markers p-p65 and TNF-α in the MMC-cata group were significantly lower than those in the CP and siRNA3 groups, whereas the expression levels of these factors in the siRNA3 group were slightly lower or similar to those in the CP group (Fig. 5J, K). Additionally, compared with the MMC-cata group, the siRNA3 group presented significantly lower GSH and T-SOD levels (Fig. 5L, M) and significantly higher MDA levels (Fig. 5N). Western blotting also revealed that the expression levels of the necroptosis markers RIP, RIP3, and MLKL, as well as their phosphorylated forms, were significantly lower in the MMC-cata group than in the CP and siRNA3 groups, with no difference between the siRNA3 and CP groups (Fig. 5O, P). Immunohistochemical staining revealed that compared with that in the CP and siRNA3 groups, MLKL expression in the MMC-cata group was significantly lower (Fig. 5Q–T). These findings suggest that knockdown of the VEGF-A gene abolishes the enhanced protective effect of catalpol-pretreated MMCs against cisplatin-induced AKI.

### Neutralizing VEGF-A or blocking VEGFR2 abolishes the ability of catalpol pretreatment to enhance the efficacy of MMCs

**Fig. 6:**
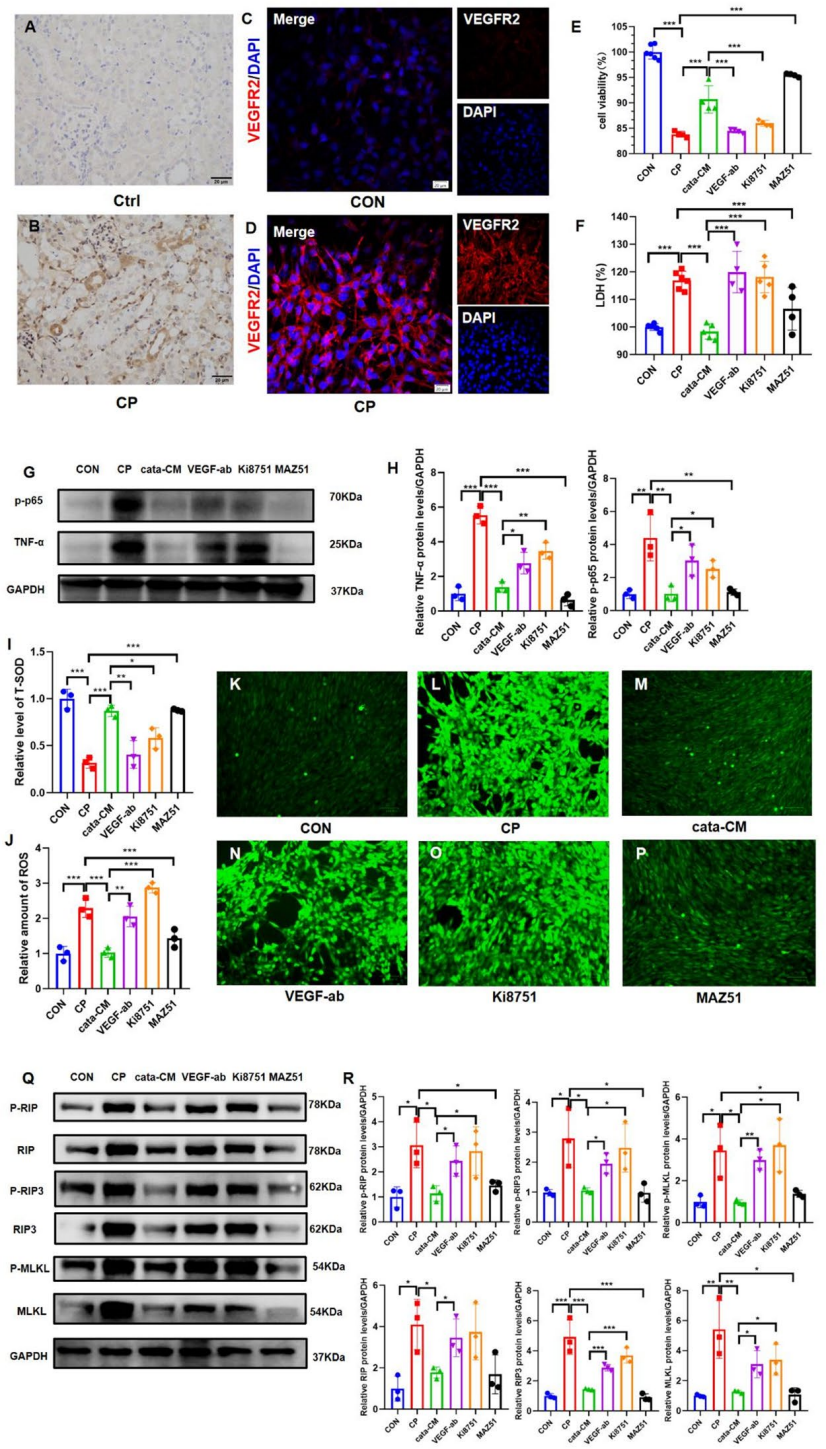
Neutralizing VEGF-A or Blocking VEGFR2 Abrogates the Protective Effect of Catalpol-Pretreated MMCs in vitro. (**A**, B) Immunohistochemical staining images showing the expression of VEGFR2 in the kidneys of mice from different groups (400×). (**C**, **D**) Immunofluorescence staining images showing the expression of VEGFR2 in TCMK-1 cells from different groups (400×). (**E**, **F**) CCK-8 and LDH assay results showing the proliferative activity and cytotoxicity of TCMK-1 cells in different groups (*n* = 6). (**G**, **H**) Western blot showing the expression of the inflammatory markers p-p65 and TNF-α in cells from different groups and statistical analysis (*n* = 3). (**I**-**P**) Levels of the oxidative stress indicators ROS and T-SOD and ROS staining in TCMK-1 cells (*n* = 3). (**Q**-**R**) Western blot showing the expression of the necroptosis markers RIP, RIP3, and MLKL, as well as their phosphorylated forms, in cells from different groups and statistical analysis (*n* = 3). *CON*: control group; *CP*: cisplatin injury group; *cata-CM*: catalpol-pretreated MMC supernatant group; *VEGF-ab*: catalpol-pretreated MMC supernatant + VEGF-A neutralizing antibody group; Ki8751: catalpol-pretreated MMC supernatant + VEGFR2 inhibitor Ki8751 group; MAZ51: catalpol-pretreated MMC supernatant + VEGFR3 inhibitor MAZ51 group. ***P < 0.001, **P < 0.01, *P < 0.05

To verify whether VEGF-A acts as an effector molecule in the enhanced therapeutic efficacy of catalpol-pretreated MMCs and to identify its receptor, we employed two approaches: neutralizing VEGF-A and blocking the receptor through which VEGF-A exerts its effects. Since VEGFR2 is the primary receptor for VEGF-A [46], we examined the expression of VEGFR2 in the kidneys of cisplatin-injured mice and TCMK-1 cells. Immunohistochemical staining revealed increased expression of VEGFR2 in injured renal tubules (Fig. 6A, B), and immunofluorescence staining revealed significantly greater expression of VEGFR2 in cisplatin-injured TCMK-1 cells than in control cells (Fig. 6C, D). In vitro experiments demonstrated that the addition of 0.1 µg/ml VEGF-A neutralizing antibody (VEGF-ab) or 10 nM VEGFR2 inhibitor (Ki8751) neutralized the protective effect of the catalpol-pretreated MMC supernatant on TCMK-1 cells, resulting in decreased cell viability and increased intracellular LDH levels (Fig. 6E, F). Western blot analysis revealed that the expression levels of the inflammatory markers p-p65 and TNF-α in the VEGF-ab and Ki8751 groups were greater than those in the cata-CM group (Fig. 6G, H). Compared with the cata-CM group, the VEGF-ab and Ki8751 groups exhibited reduced T-SOD activity (Fig. 6I), increased ROS levels (Fig. 6J–P), and increased expression of the necroptosis markers RIP, RIP3, and MLKL, as well as their phosphorylated forms (Fig. 6Q, R). In contrast, the addition of 5 µM MAZ51, a VEGFR3 inhibitor, had no effect on the therapeutic efficacy of cata-CM. These findings suggest that inhibition of the VEGF-A/VEGFR2 axis abolishes the enhanced therapeutic effect of catalpol-pretreated MMCs.

### Application of VEGF-A alone alleviates cisplatin-induced renal tubular epithelial cell injury

**Fig. 7:**
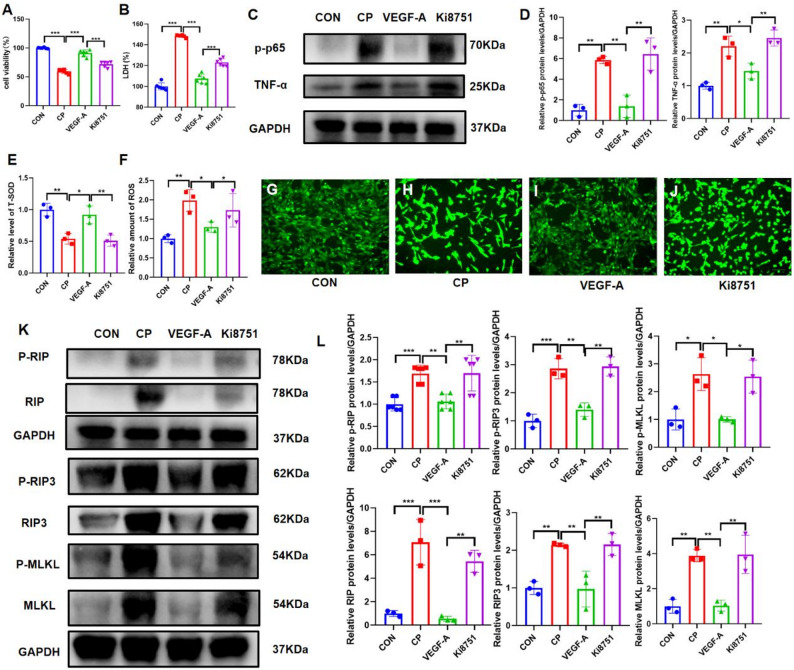
Application of VEGF-A Alone Alleviates Cisplatin-Induced Renal Tubular Epithelial Cell Injury. (**A**, **B**) CCK-8 and LDH assay results showing the proliferative activity and cytotoxicity of TCMK-1 cells in different groups (*n* = 6). (**C**, **D**) Western blot showing the expression of the inflammatory markers p-p65 and TNF-α in cells from different groups and statistical analysis (*n* = 3). (**E**-**J**) The levels of the oxidative stress indicators ROS and T-SOD and ROS staining in TCMK-1 cells (*n* = 3). (**K**, **L**) Western blot showing the expression of the necroptosis markers RIP, RIP3, and MLKL, as well as their phosphorylated forms, in cells from different groups and statistical analysis (*n* = 3). *CON* control group; *CP* cisplatin injury group; *VEGF-A* VEGF-A treatment group; Ki8751: VEGF-A + VEGFR2 inhibitor Ki8751 group. ***P < 0.001, **P < 0.01, *P < 0.05

To determine whether VEGF-A is the effector molecule responsible for the therapeutic effects of MMCs-cata, 5 ng/ml VEGF-A alone was used to treat cisplatin-injured TCMK-1 cells. VEGF-A treatment alone also alleviated cisplatin-induced TCMK-1 cell injury, as evidenced by increased cell viability and decreased intracellular LDH levels (Fig. 7A, B). Western blot analysis revealed that the expression levels of the inflammatory markers p-p65 and TNF-α were lower in the VEGF-A group than in the CP group (Fig. 7C, D). Additionally, T-SOD activity increased and ROS levels decreased in the VEGF-A group (Fig. 7E–J). Western blotting demonstrated that the expression levels of the necroptosis markers RIP, RIP3, and MLKL, as well as their phosphorylated forms, were lower in the VEGF-A group than in the CP group (Fig. 7K, L). The therapeutic effect of VEGF-A was abolished upon the addition of the VEGFR2 inhibitor Ki8751, indicating that VEGF-A alone can alleviate cisplatin-induced renal tubular epithelial cell injury. These findings suggest that VEGF-A may be the effector molecule through which MMCs-cata exert their enhanced protective effects against cisplatin-induced injury.

### The protective effect of catalpol-pretreated MMCs May be related to the Inhibition of STAT3 activity and activation of the p38 pathway

**Fig. 8:**
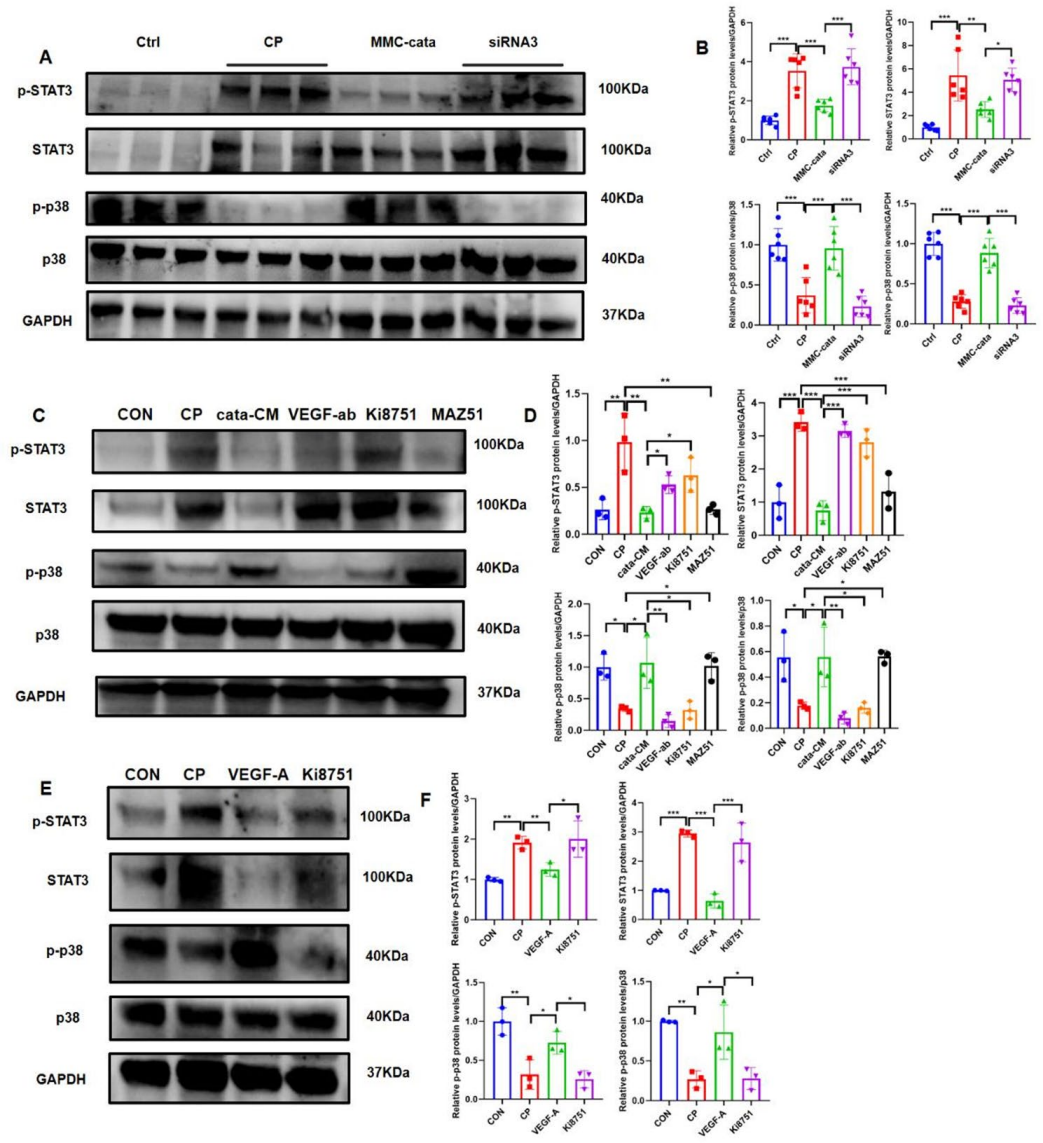
Pretreatment of MMCs with catalpol may suppress the STAT3 pathway and activate the p38 pathway. (**A**, **B**) Western blot showing the expression of STAT3, p38, and their phosphorylated proteins in kidney tissues from mice in different groups (*n* = 6). Ctrl: control group; CP: cisplatin model group; MMC-cata: catalpol-pretreated MMC treatment group; siRNA3: siRNA3-transfected MMC treatment group. (**C**, **D**) Western blot showing the expression of STAT3, p38, and their phosphorylated proteins in cells from different groups (*n* = 3). CON: control group; CP: cisplatin injury group; cata-CM: catalpol-pretreated MMC supernatant group; VEGF-ab: catalpol-pretreated MMC supernatant + VEGF-A neutralizing antibody group; Ki8751: catalpol-pretreated MMC supernatant + VEGFR2 inhibitor Ki8751 group; MAZ51: catalpol-pretreated MMC supernatant + VEGFR3 inhibitor MAZ51 group. (**E**–**F**) Western blot showing the expression of STAT3, p38, and their phosphorylated proteins in cells from different groups (*n* = 3). CON: control group; CP: cisplatin injury group; VEGF-A: VEGF-A treatment group; and Ki8751: VEGF-A + VEGFR2 inhibitor Ki8751 group. ***P < 0.001, **P < 0.01, *P < 0.05

The RNA sequencing results of cisplatin-injured renal tubular epithelial cells suggested that cisplatin injury might suppress the MAPK pathway and increase STAT phosphorylation (Supplementary Fig S1B). Therefore, we investigated these two pathways. Western blot revealed that compared with the other groups, the cisplatin injury group and the siRNA3 group exhibited increased expression of STAT3 and its phosphorylated proteins, along with decreased phosphorylation of p38. In contrast, the MMC-cata group exhibited the reverse trend (Fig. 8A, B). In vitro experiments revealed that cisplatin-injured TCMK-1 cells also presented increased expression of STAT3 and its phosphorylated proteins, along with decreased p38 phosphorylation. However, the addition of catalpol-pretreated MMC-conditioned medium (cata-CM) or VEGF-A reduced the expression of STAT3 and its phosphorylated proteins while increasing p38 phosphorylation. These effects were reversed upon the addition of a VEGF-A neutralizing antibody (VEGF-ab) or the VEGFR2-specific inhibitor Ki8751 (Fig. 8C–F). These findings suggest that the enhanced protective effect of catalpol-pretreated MMCs against cisplatin-induced injury may be associated with suppression of the STAT3 pathway and activation of the p38 pathway.

### Catalpol promotes VEGF-A synthesis in MMCs by activating the canonical Wnt pathway by binding to Wnt3A

**Fig. 9:**
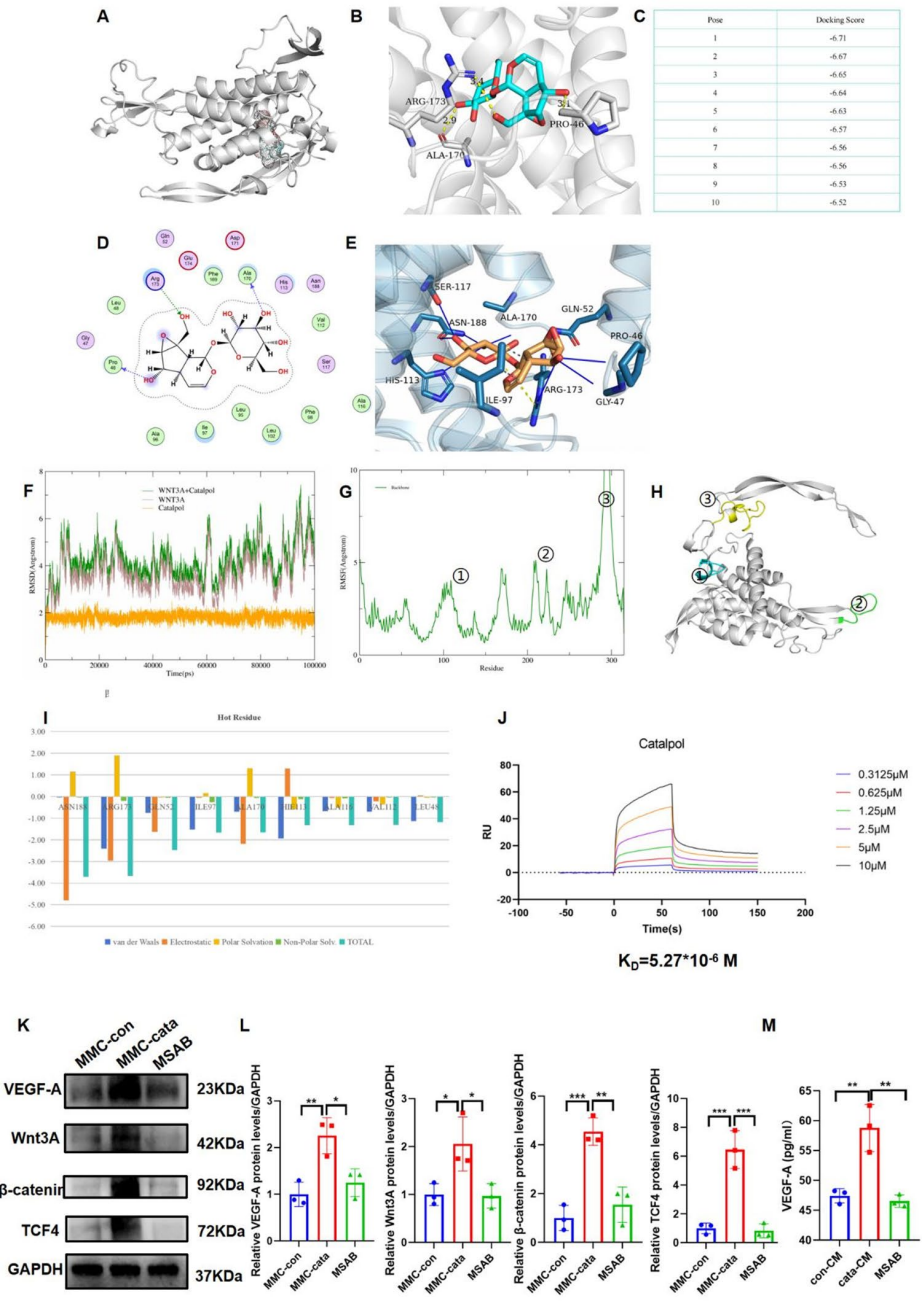
Catalpol binds to Wnt3A, activating the canonical Wnt pathway in MMCs and promoting VEGF-A secretion. (**A**, **B**) Molecular docking images of catalpol and Wnt3A. (**C**) Docking score of the interaction between catalpol and Wnt3A. (**D**, **E**) Interaction sites of catalpol and Wnt3A. (**F**) RMSDs of the molecular dynamics simulations of catalpol with the Wnt3A protein. (**G**, H) RMSFs of the molecular dynamics simulations of catalpol and the Wnt3A protein; the numerical sequence represents different binding sites. (**I**) Free energy decomposition of different binding sites of catalpol to the Wnt3A protein. (**J**) Surface plasmon resonance was used to precisely measure the binding affinity and binding mode of catalpol to Wnt3A. (**K**, **L**) Western blot showing the expression levels of VEGF-A and Wnt3A, β-catenin, and TCF-4 in MMCs treated with different drugs (*n* = 3). (M) VEGF-A content in the conditioned medium of MMCs under different drug treatments (*n* = 3). Groups in panels J-K: MMC-con: normal MMCs; MMC-cata: catalpol pretreated MMCs; MSAB: catalpol- + canonical Wnt pathway blocker MSAB-pretreated MMCs. Groups in panel L: Con-CM: normal MMC-conditioned medium; cata-CM: catalpol-pretreated MMC-conditioned medium; MSAB: catalpol- + canonical Wnt pathway blocker MSAB-pretreated MMC-conditioned medium. ***P < 0.001, **P < 0.01, *P < 0.05

Our previous studies revealed that catalpol can activate the canonical Wnt pathway in MMCs to promote epithelial differentiation. Therefore, we used computer simulations and experimental validation to investigate whether catalpol promotes VEGF-A secretion by MMCs through activation of the canonical Wnt pathway. Molecular docking revealed that catalpol forms hydrogen bonds with ILE97 of the Wnt3A molecule and strong electrostatic interactions with ARG173. Additionally, hydrophobic interactions were observed with amino acids such as PRO46 and GLY47 (Fig. 9A–E, Supplementary Table S1). Molecular dynamics simulations indicated that the RMSD of the complex system remained stable after initial relaxation, with deviations of the small molecule from the initial docking structure of less than 1 Å. The conformational fluctuations of the complex and the protein receptor overlapped closely, with fluctuations confined to within 4 Å (Fig. 9F). Statistical analysis of the RMSFs from the molecular dynamics simulations revealed that the overall backbone of the protein remained within 5 Å during the 100 ns simulation, with higher flexibility observed in regions with amino acids 96–106, 206–215, and 291–310 (Fig. 9G, H), indicating stable binding of the small molecule to the receptor protein. The SPR results revealed that catalpol rapidly bound to Wnt3A at different catalpol concentrations. The KD value of binding was 5.27 × 10^− 6^ M (Fig. 9J), indicating a strong binding. Western blot demonstrated that VEGF-A expression increased in MMCs stimulated with 1 µM catalpol, accompanied by activation of the canonical Wnt pathway, as evidenced by elevated expression of Wnt3A, β-Catenin, and TCF4. However, upon the addition of the canonical Wnt pathway inhibitor MSAB, this pathway was suppressed, and the expression levels of VEGF-A, Wnt3A, β-Catenin, and TCF4 decreased (Fig. 9K, L). ELISAs of the cell supernatant revealed that VEGF-A levels were lower in the MSAB group than in the catalpol group (Fig. 9M). These findings suggest that catalpol promotes VEGF-A secretion by MMCs by binding to Wnt3A and activating the canonical Wnt pathway.

## Discussion

**Fig. 10:**
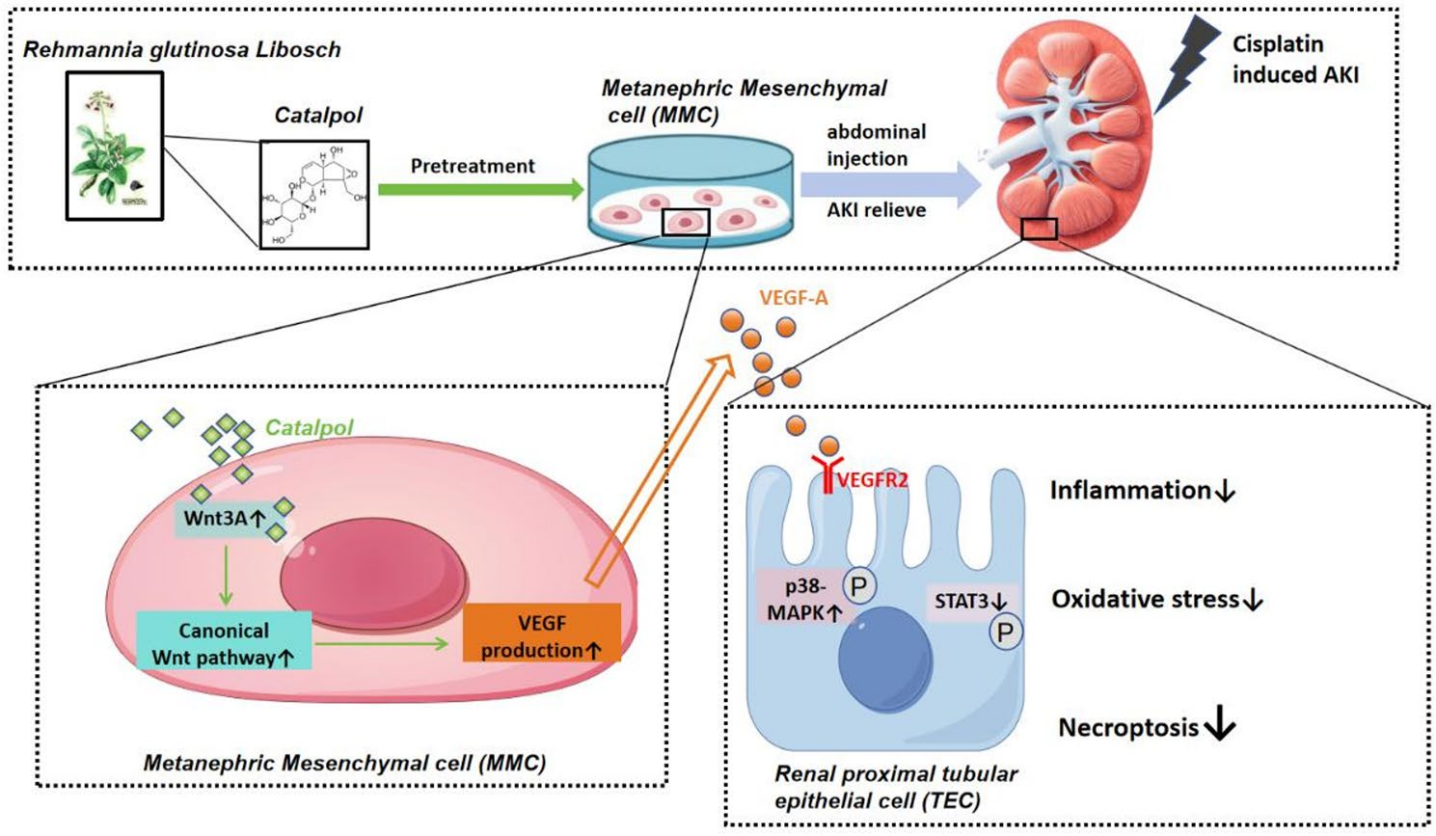
Catalpol-pretreated MMCs alleviate cisplatin-induced acute kidney injury through multiple pathways by promoting VEGF-A secretion. Catalpol-pretreated MMCs activate the canonical Wnt pathway, increasing the synthesis and secretion of VEGF-A. VEGF-A acts on VEGFR2 in renal tubular epithelial cells, mitigating inflammation, oxidative stress, and necroptosis by activating the p38 signaling pathway and suppressing STAT3 signaling, thereby alleviating cisplatin-induced acute kidney injury

This study innovatively employs catalpol, an active monomer derived from the kidney-tonifying and essence-replenishing traditional Chinese medicine *Rehmannia glutinosa*, to precondition metanephric mesenchymal cells (MMCs). For the first time, it has been revealed that catalpol can activate the canonical Wnt pathway in MMCs, increase their secretion of VEGF-A, and act on VEGFR2 in renal tubular epithelial cells. This process may involve activation of the p38 signaling pathway and the suppression of STAT3 signaling, thereby reducing inflammation, oxidative stress, and necroptosis and mitigating cisplatin-induced acute kidney injury (Fig. 10). This research offers a novel approach for integrating traditional Chinese and Western medicines for the prevention and treatment of acute kidney injury.

This study revealed that intraperitoneal injection of catalpol-pretreated MMCs more effectively alleviated cisplatin-induced acute kidney injury, mainly by reducing necroptosis. The mechanisms underlying cisplatin-induced acute kidney injury are complex and involve inflammation, oxidative stress, and other pathways. Recent studies have reported that necroptosis may play a major role in cisplatin-induced acute renal tubular necrosis [26, 27]. In this study, necroptosis-related genes and pathways were activated in cisplatin-treated proximal tubular epithelial cells both in vivo and in vitro. This study utilized catalpol-pretreated MMCs and found that such pretreatment improved therapeutic outcomes for cisplatin-induced acute kidney injury. Both animal and cell models have demonstrated that catalpol-pretreated MMCs alleviate injury primarily by reducing inflammation, oxidative stress, and necroptosis. Compared with the model group, both the catalpol-pretreated MMC group and the regular MMC group exhibited mitigated inflammation, oxidative stress, and necroptosis. However, there was little difference between these two groups in terms of reducing inflammation and oxidative stress, while compared with regular MMCs, catalpol-pretreated MMCs significantly reduced necroptosis, thereby alleviating kidney and tubular epithelial cell injury. These findings confirm the critical role of necroptosis in acute kidney injury.

Does the therapeutic effect of catalpol-pretreated MMCs result from its direct differentiation and replacement of damaged cells or from the secretion of cytokines to mitigate injury? Previous studies have reported that pretreatment of stem/progenitor cells with small-molecule compounds from traditional Chinese medicine can increase cell activity, promote paracrine effects, and facilitate differentiation into kidney-specific cells, thereby improving therapeutic efficacy against acute kidney injury [28]. Metanephric mesenchymal cells (MMCs) originate from the metanephric stage of kidney development and contain multipotent renal progenitor cells [29] Theoretically, MMCs can migrate to injury sites and differentiate into kidney-specific structures. Although the literature reports that intravenously injected mesenchymal stem cells can home to injured kidneys, their therapeutic effects are likely mediated primarily by paracrine actions [30]. In this study, RNA sequencing of catalpol-pretreated MMCs revealed the activation of pathways related to stem cell differentiation and VEGF synthesis, indicating that both mechanisms may contribute to their therapeutic effects. To determine whether the therapeutic effect is related to MMC differentiation and the replacement of damaged tubular epithelial cells or to the secretion of cytokines, we fluorescently labeled MMCs and performed in vivo fluorescence tracing. Live imaging revealed that the MMCs primarily remained in situ after intraperitoneal injection. In vitro experiments confirmed that the supernatant of catalpol-pretreated MMCs contained increased levels of VEGF-A and that the supernatant alone could alleviate cisplatin-induced tubular epithelial cell injury. Therefore, catalpol-pretreated MMCs primarily exert therapeutic effects by secreting cytokines after intraperitoneal injection.

Is VEGF-A the effector molecule through which catalpol-pretreated MMC exerts its therapeutic effects? Vascular endothelial growth factor (VEGF) is a growth factor that has significant proangiogenic activity [31], promotes mitosis and inhibits apoptosis in endothelial cells, increases vascular permeability, and enhances cell migration [32]. VEGF and its receptors (VEGFRs) are expressed not only on endothelial cells but also on nonendothelial cells [33, 34]. VEGF plays a crucial role in kidney development, promoting tubulogenesis and angiogenesis [35]. Anti-VEGF therapies for tumors often lead to acute kidney injury [36]. Decreased VEGF gene expression in kidney tissues is associated with aggravated acute kidney injury [37]. Catalpol pretreatment of bone marrow mesenchymal stem cells promotes VEGF secretion [38]. Considering that VEGF-related pathways were activated in catalpol-pretreated MMCs in this study, we focused on exploring VEGF-related pathways. We first measured VEGF-A levels in catalpol-pretreated MMCs and their supernatants. Using four approaches—knocking down the VEGF-A gene, adding VEGF-A neutralizing antibodies, blocking VEGFR2, and applying VEGF-A alone—we validated the role of VEGF-A in cisplatin-induced AKI. The results revealed increased VEGF-A levels in catalpol-pretreated MMCs and their supernatants. The protective effects of catalpol-pretreated MMCs and their supernatant were neutralized by knocking down VEGF-A, adding anti-VEGF-A neutralizing antibodies, or blocking VEGFR2, while blocking VEGFR3 had no effect. The application of VEGF-A alone also alleviated injury, and its protective effects were neutralized by blocking VEGFR2, confirming that VEGF-A is the effector molecule that primarily exerts its protective effects through VEGFR2.

How does VEGF-A exert its effects after it acts on VEGFR2, and what are the downstream pathways involved? In this study, sequencing of cisplatin-injured proximal tubular epithelial cells revealed that MAPK signaling was suppressed in the injury group, whereas biological processes related to STAT protein phosphorylation were activated. The mitogen-activated protein kinase (MAPK) pathway is involved in various biological processes, such as cell proliferation, differentiation, migration, and apoptosis. In acute kidney injury (AKI), the MAPK pathway plays a dual role: on the one hand, it may activate inflammation and induce cell damage, and on the other hand, it is associated with regulating tissue-resident stem cells, maintaining homeostasis, and promoting kidney repair mechanisms [39]. The JAK/STAT pathway is another important intracellular signaling pathway that primarily regulates cell growth, differentiation, immune responses, and inflammation. In AKI, the JAK/STAT pathway plays a significant role in enhancing inflammatory responses, promoting fibrosis, and exacerbating kidney injury [40, 41]. This study revealed that activation of the p38/MAPK pathway and suppression of the STAT pathway may be related to alleviating cisplatin-induced AKI. Therefore, VEGF-A can alleviate cisplatin-induced AKI by activating p38 signaling and suppressing STAT3 signaling.

How does catalpol interact with MMCs to promote VEGF-A secretion? Catalpol, a flavonoid glycoside derived from Rehmannia, is widely used to treat diabetes, osteoporosis, atherosclerosis, and Alzheimer’s disease [42]. Its therapeutic effects may be related to its anti-inflammatory, antioxidant, insulin resistance reducing, and cytoprotective properties [43–45]. There are also reports on the use of catalpol pretreatment to enhance the therapeutic efficacy of stem cells. For instance, Ju et al. reported that pretreatment of bone marrow mesenchymal stem cells (BMSCs) with catalpol improved their tolerance to hypoxia and glucose deprivation, promoted VEGF secretion, and enhanced therapeutic efficacy in a rat model of myocardial infarction [38]. However, this study did not identify the receptor through which catalpol exerts its effects or the receptor through which VEGF-A mediates its therapeutic effects downstream. Our previous research revealed that catalpol activates the canonical Wnt pathway to promote the differentiation of MMCs into epithelial cells. Therefore, we chose the Wnt pathway for further investigation. Molecular docking and molecular dynamics experiments demonstrated that catalpol binds well to Wnt3A and forms stable interactions. Western blot revealed that the canonical Wnt pathway was activated, and blocking this pathway reduced VEGF-A expression in MMCs. By binding to Wnt3A in MMCs, catalpol activates the canonical Wnt pathway, thereby promoting VEGF-A secretion.

However, our study has several limitations. Owing to the multitarget nature of small-molecule compounds in traditional Chinese medicine, VEGF-A is likely not the only effector molecule stimulated by catalpol in MMCs, and this study did not identify other potential effector molecules or protective mechanisms. This study solely investigated the efficacy and mechanisms of catalpol-pretreated MMCs in treating AKI but lacked long-term observational assessments regarding the safety of MMC therapy for AKI, such as tumorigenicity and the risk of viral infection.

## Conclusion

In summary, our research demonstrates that the renoprotective effect of catalpol-pretreated MMCs occurs through the activation of the canonical Wnt pathway, promoting the secretion of VEGF-A, which acts on the VEGFR2 receptor, activates downstream p38-MAPK pathways, and inhibits JAK-STAT pathway activation. This multifaceted approach reduces inflammation, oxidative stress, and necroptosis, leading to the observed therapeutic effects. This research offers a novel approach for the prevention and treatment of acute kidney injury by integrating traditional Chinese and Western medicines.

## Supplementary Information

Supplementary Material 1.

Supplementary Material 2.

## Abbreviations

AKI: Acute kidney injury
BUN: Blood urea nitrogen
CCK-8: Cell counting kit-8
GSH: Glutathione
LDH: lactate dehydrogenase
MAPK: Mitogen-activated protein kinase
MDA: Malondialdehyde
MMC: Metanephric mesenchymal cell
RMSD: Root mean square deviation
RNA-seq: RNA sequencing
ROS: Reactive oxygen species
SCr: Serum creatinine
STAT3: Signal transducer and activator of transcription 3
T-SOD: Total superoxide dismutase
VEGF-A: Vascular endothelial growth factor-A
VEGFR2: Vascular endothelial growth factor receptor 2
