# Supplementary material for: Pretreatment of metanephric mesenchymal cells with catalpol mitigates acute kidney injury through VEGF-A secretion via multiple mechanisms
Source: Stem Cell Res Ther. 2026 Mar 28;17:175. doi: 10.1186/s13287-026-04914-9 (PMC13151338; doi:10.1186/s13287-026-04914-9)

Uncropped gels for Western Blots in Figure 1

Figure 1 J

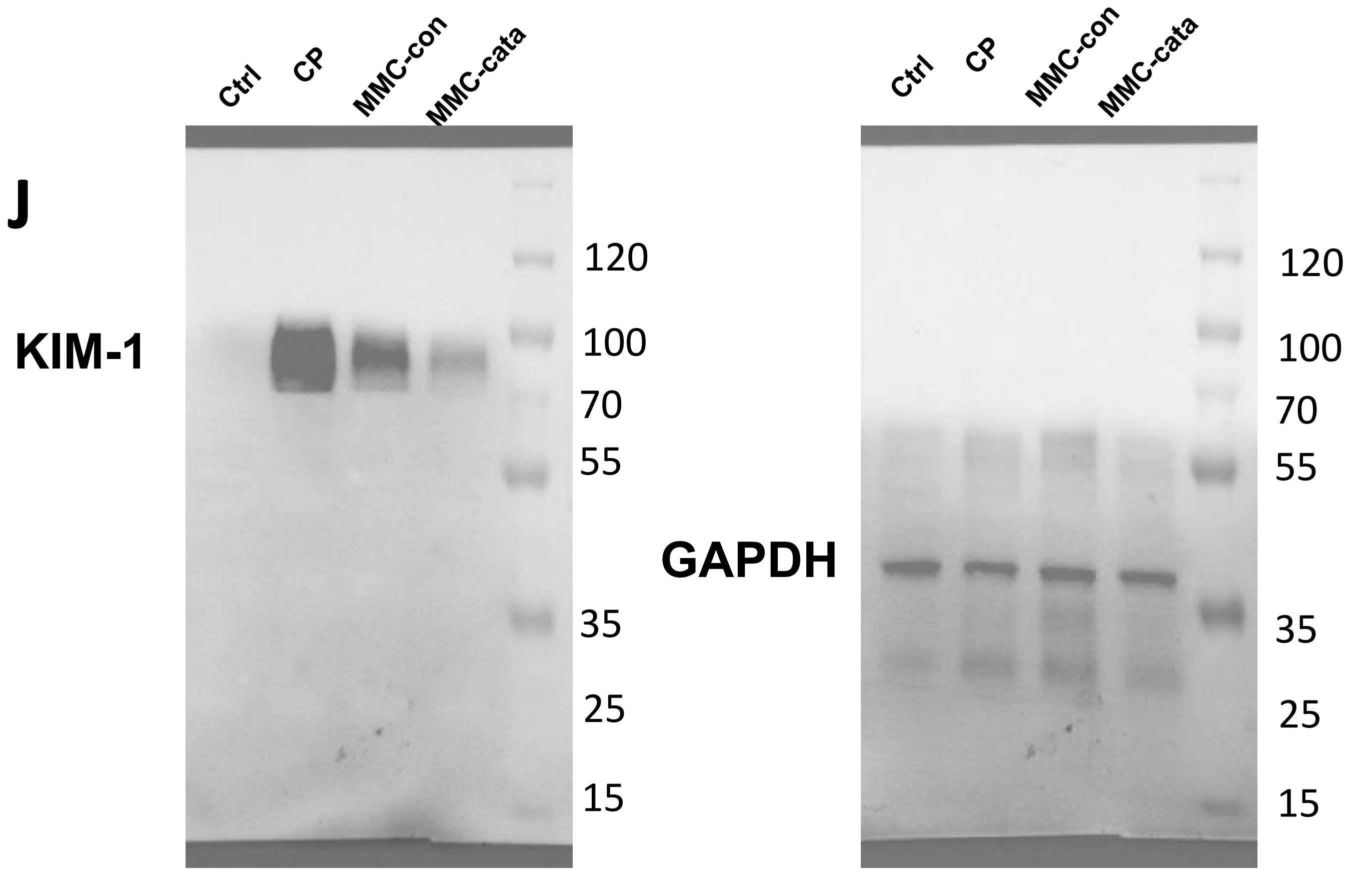

Uncropped gels for Western Blots in Figure 2

Figure 2A

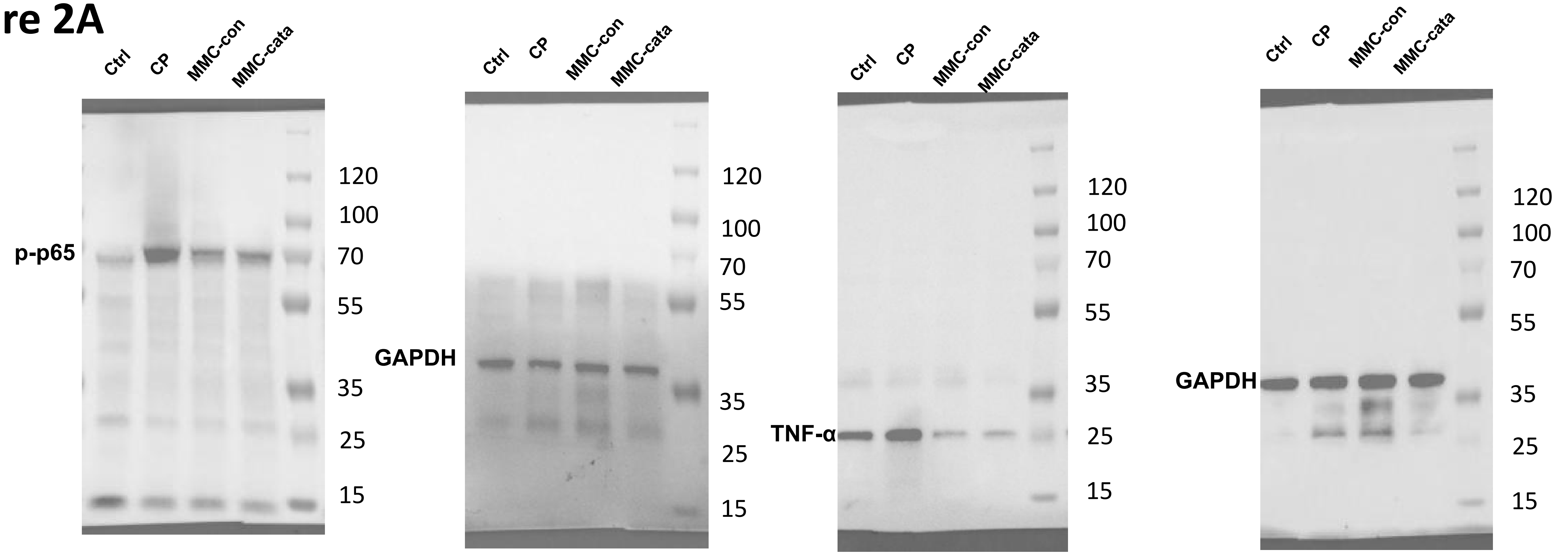

Figure 2F

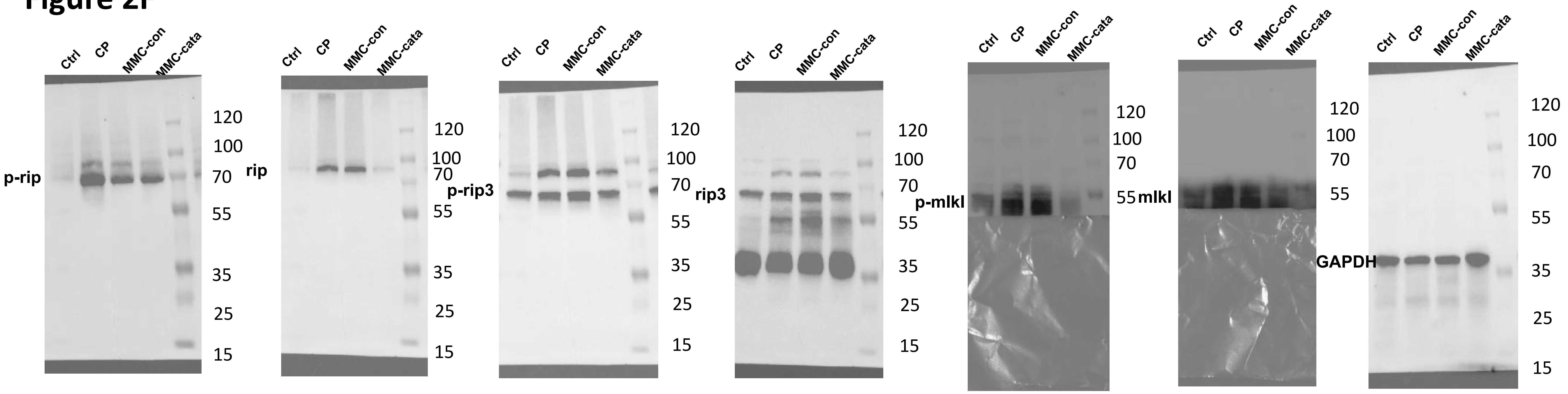

Uncropped gels for Western Blots in Figure 3

Figure 3C

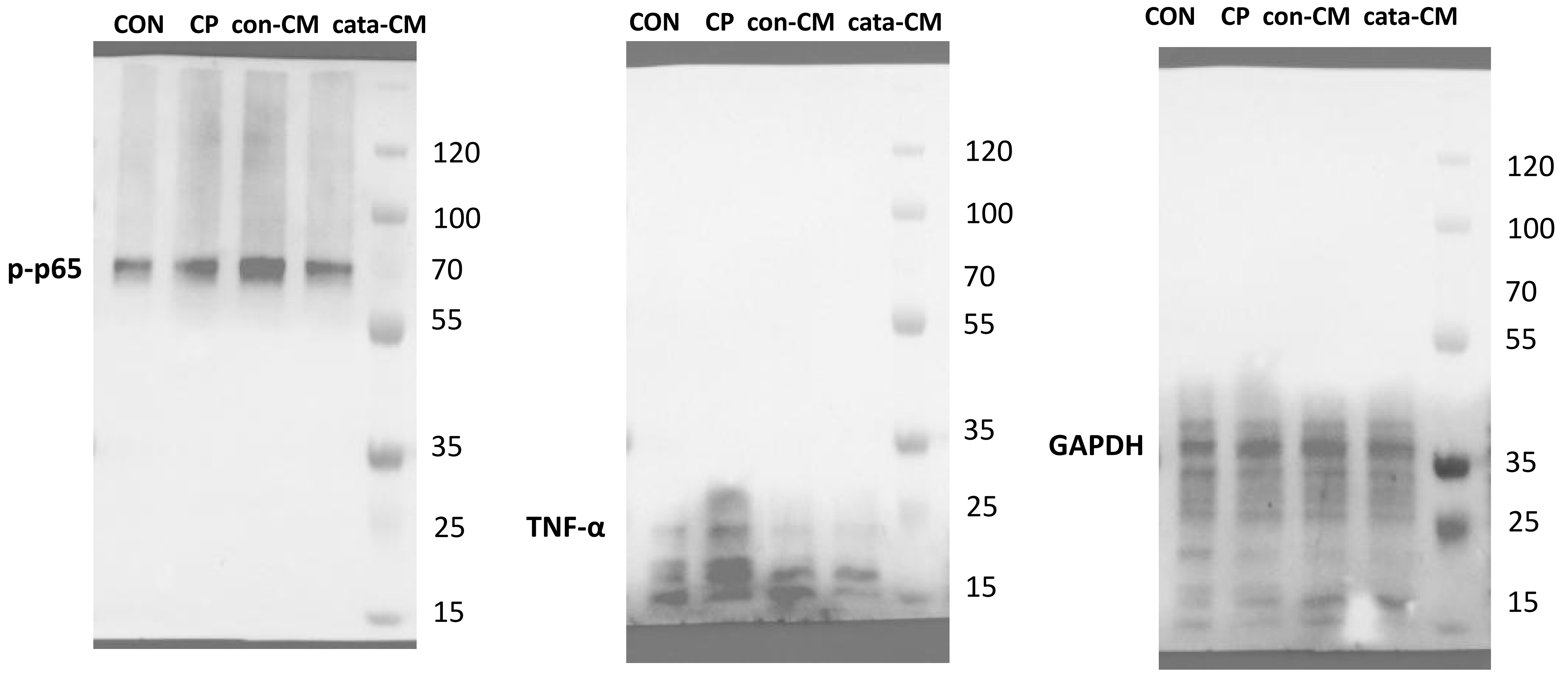

Figure 3K

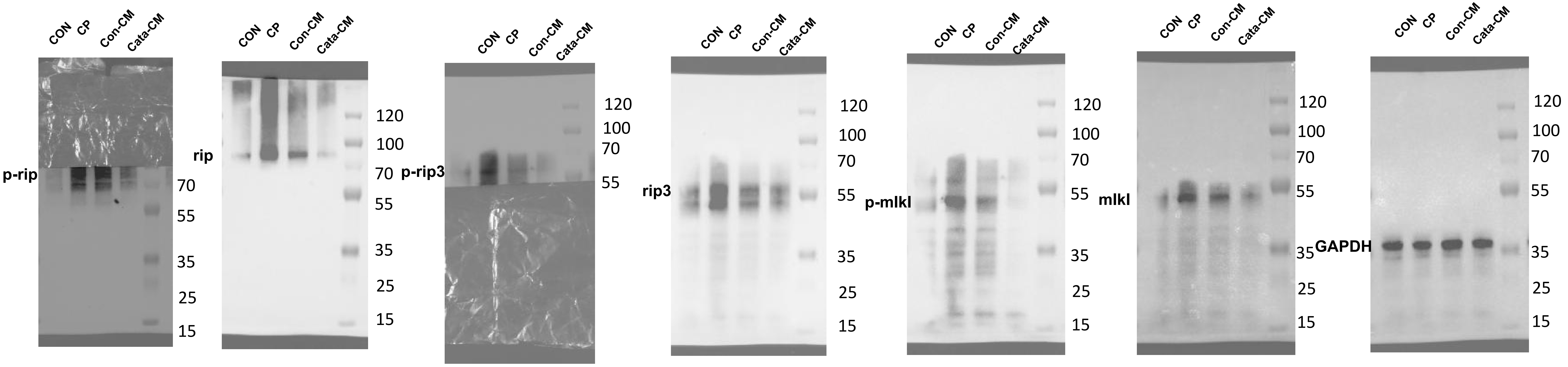

Uncropped gels for Western Blots in Figure 5

Figure 5A

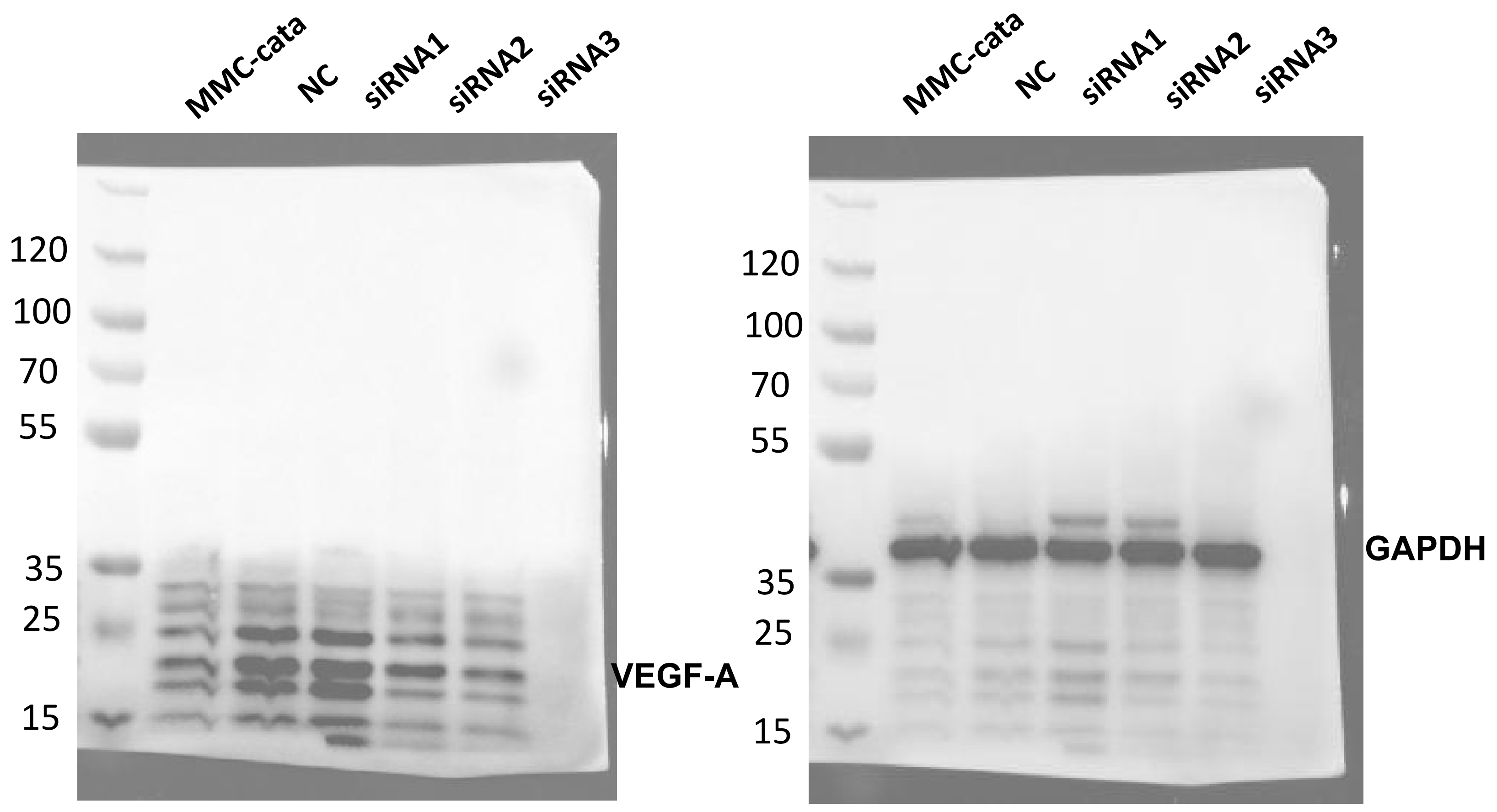

Figure 5J

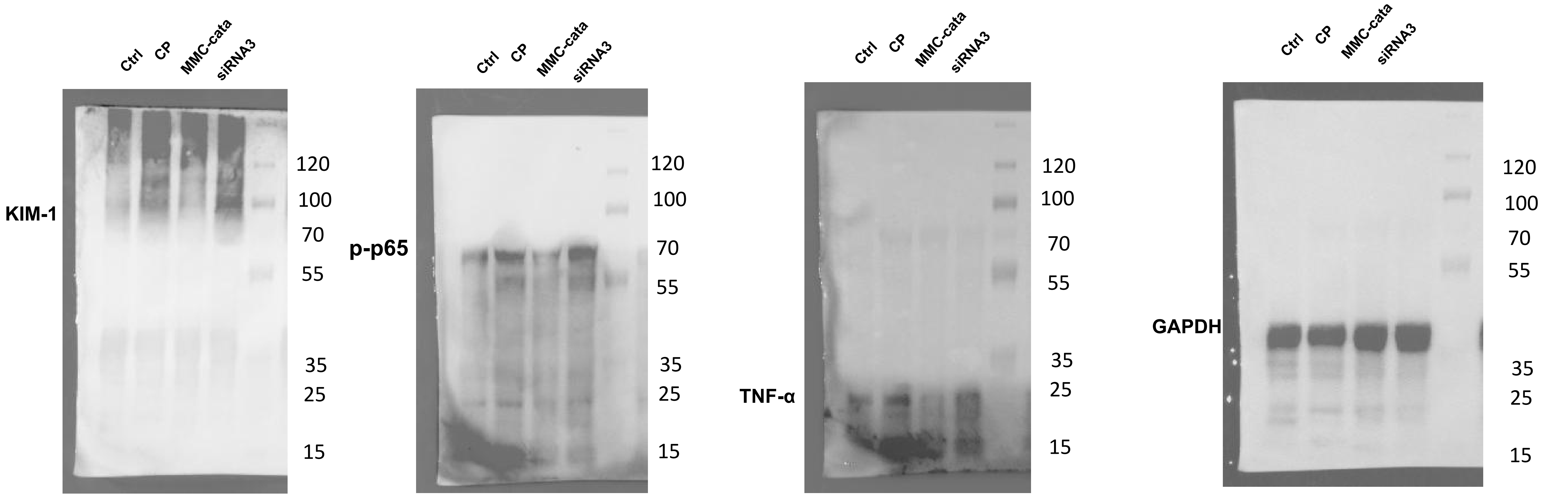

Uncropped gels for Western Blots in Figure 5

Figure 5O

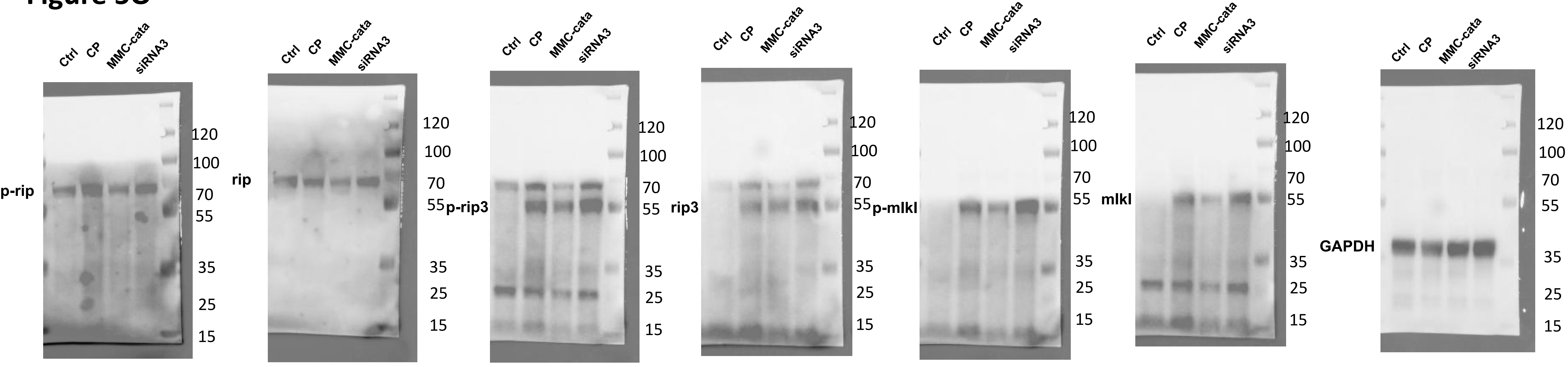

Uncropped gels for Western Blots in Figure 6

Figure 6G

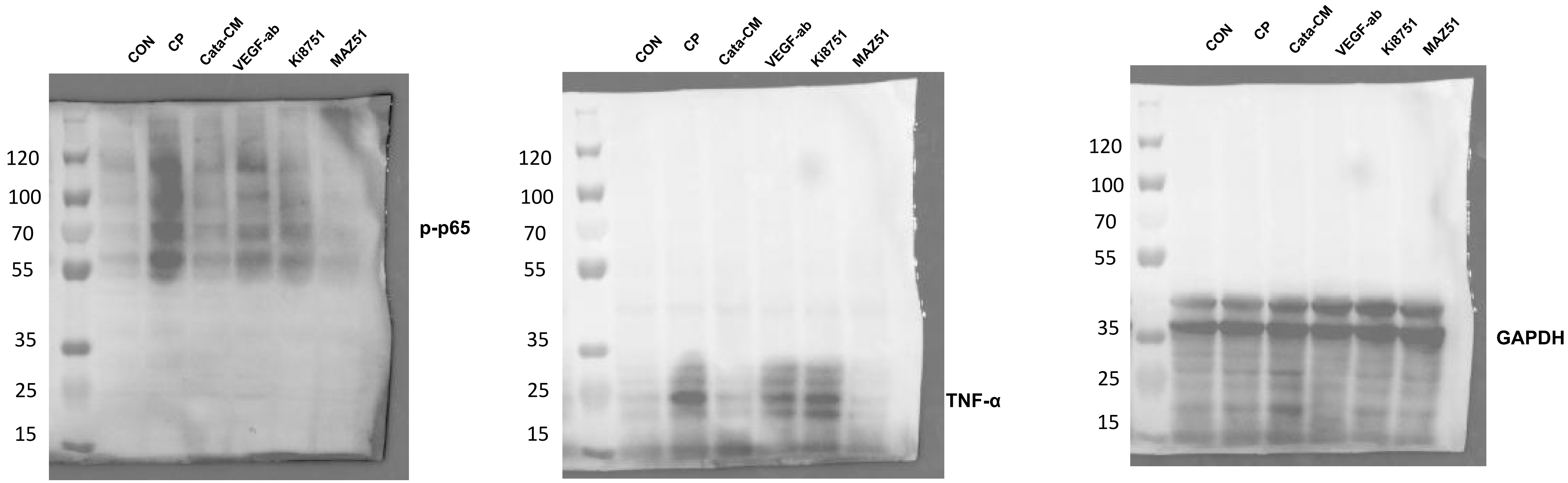

Uncropped gels for Western Blots in Figure 6

Figure 6G

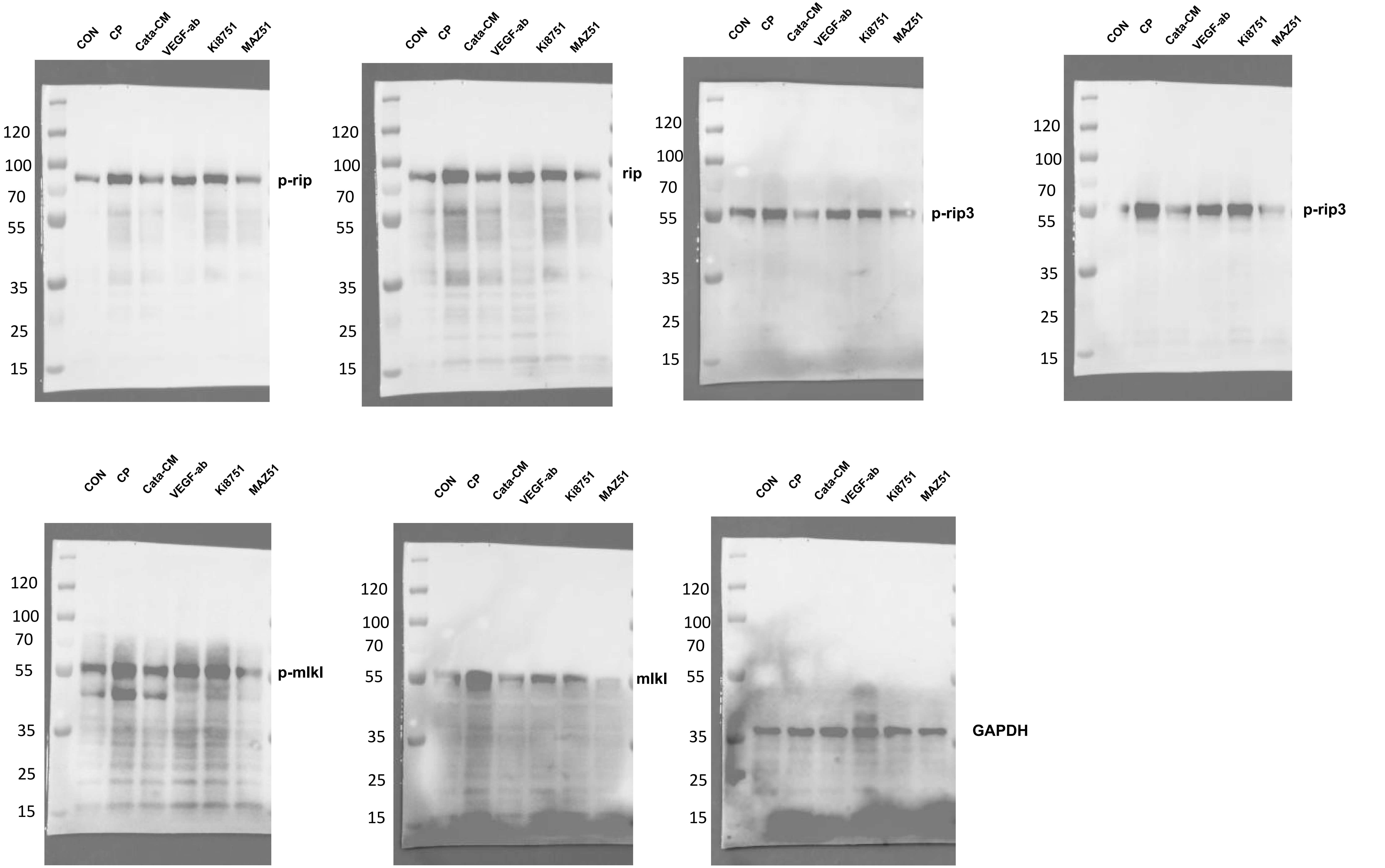

Uncropped gels for Western Blots in Figure 7

Figure 7C

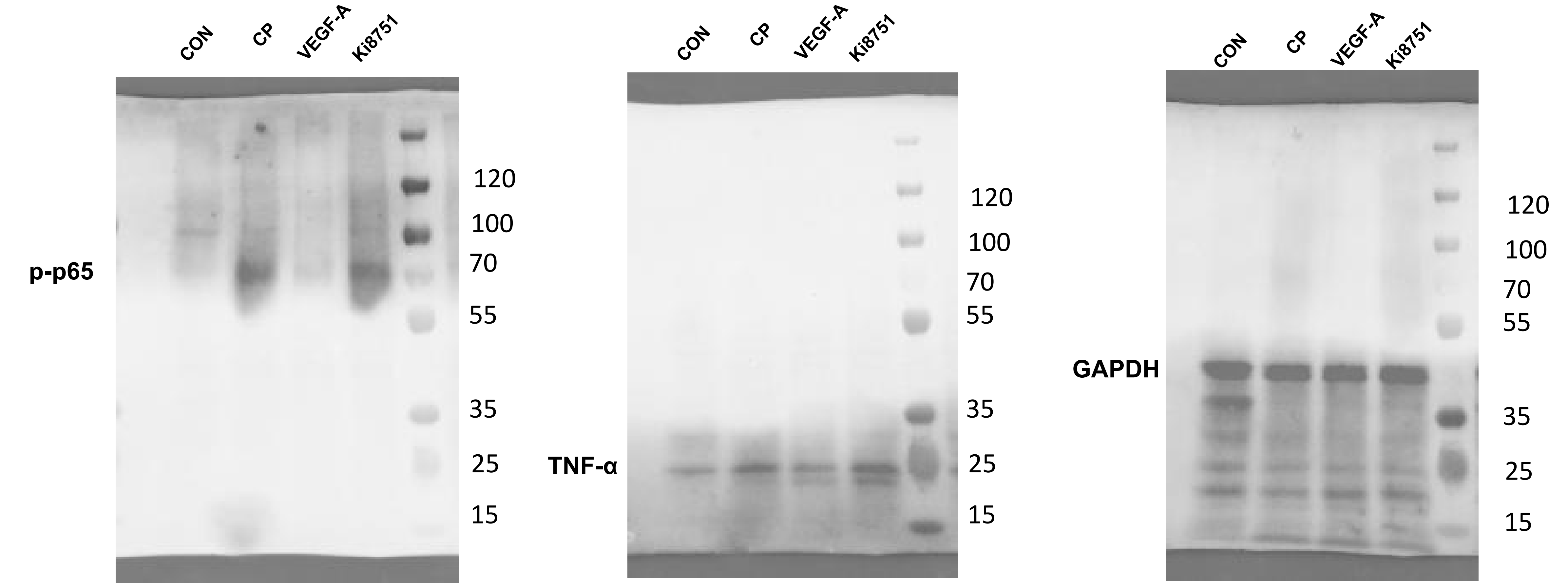

Figure 7K

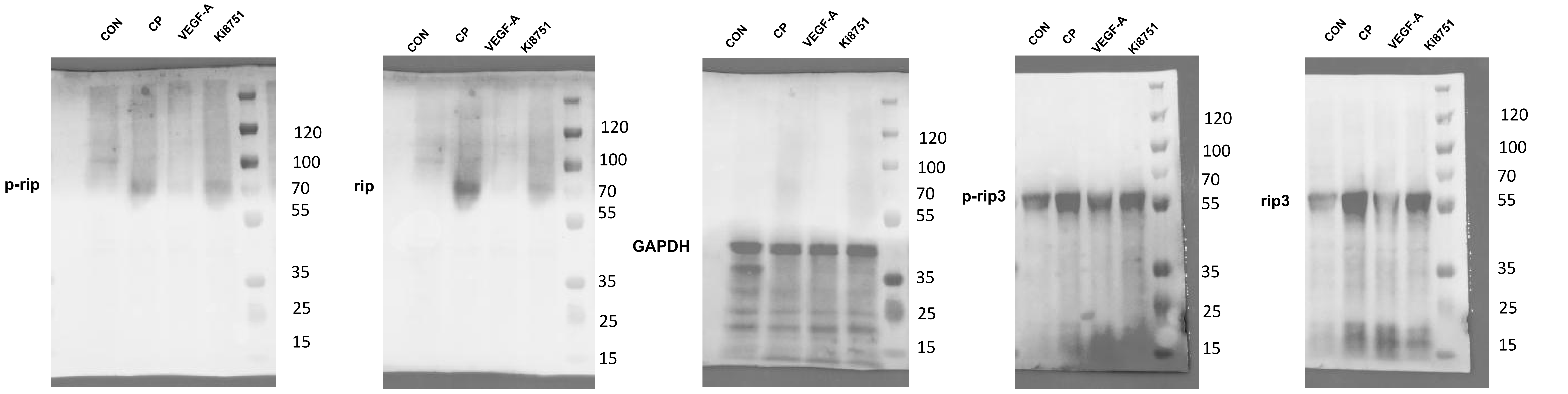

Uncropped gels for Western Blots in Figure 7

Figure 7K

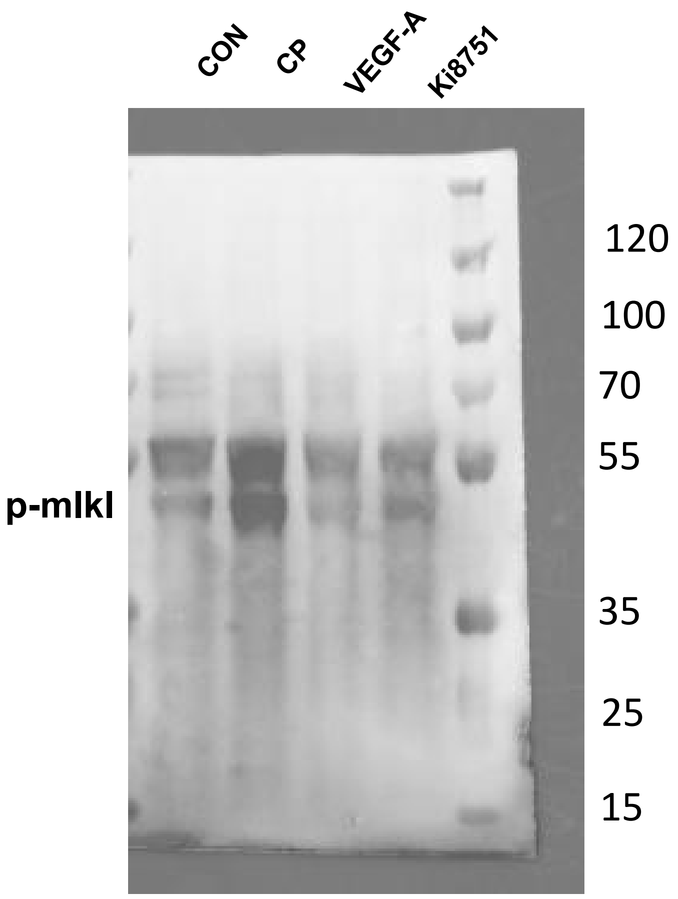

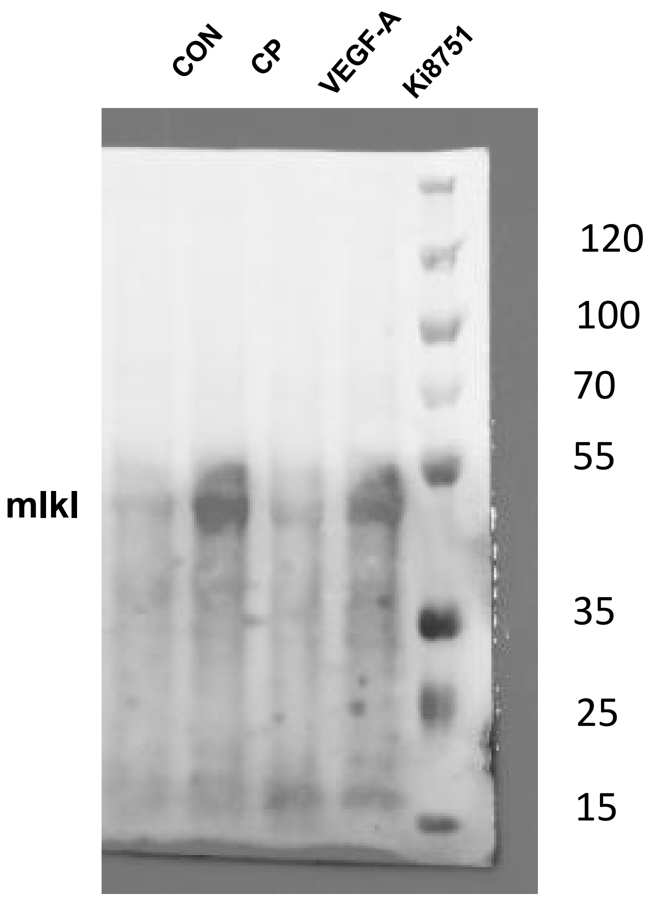

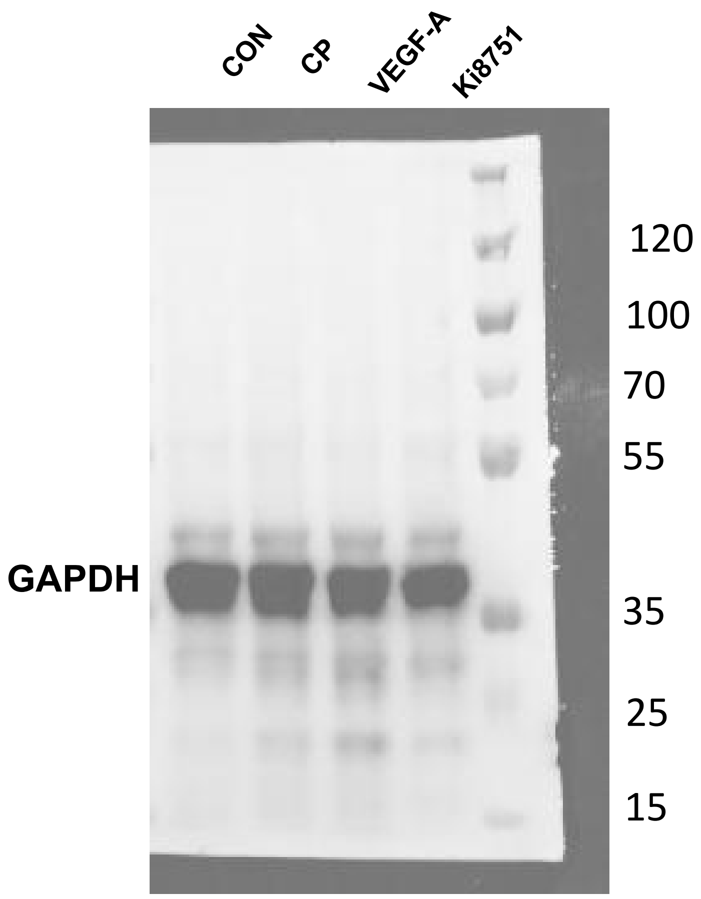

Uncropped gels for Western Blots in Figure 8

Figure 8A

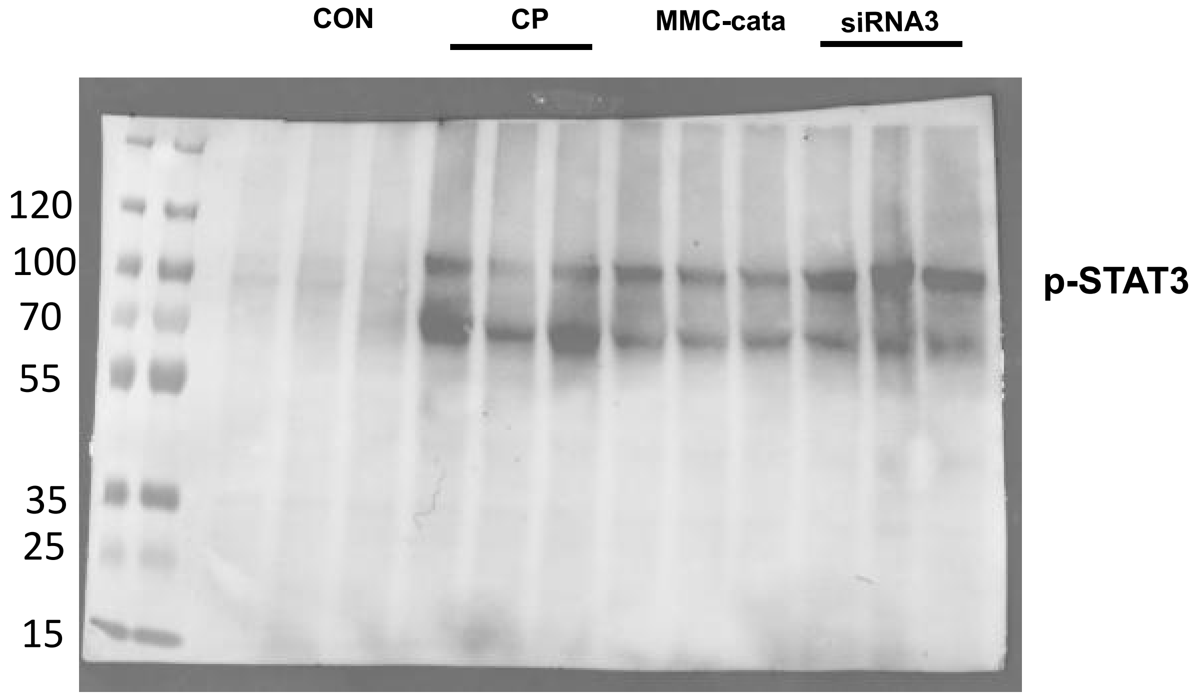

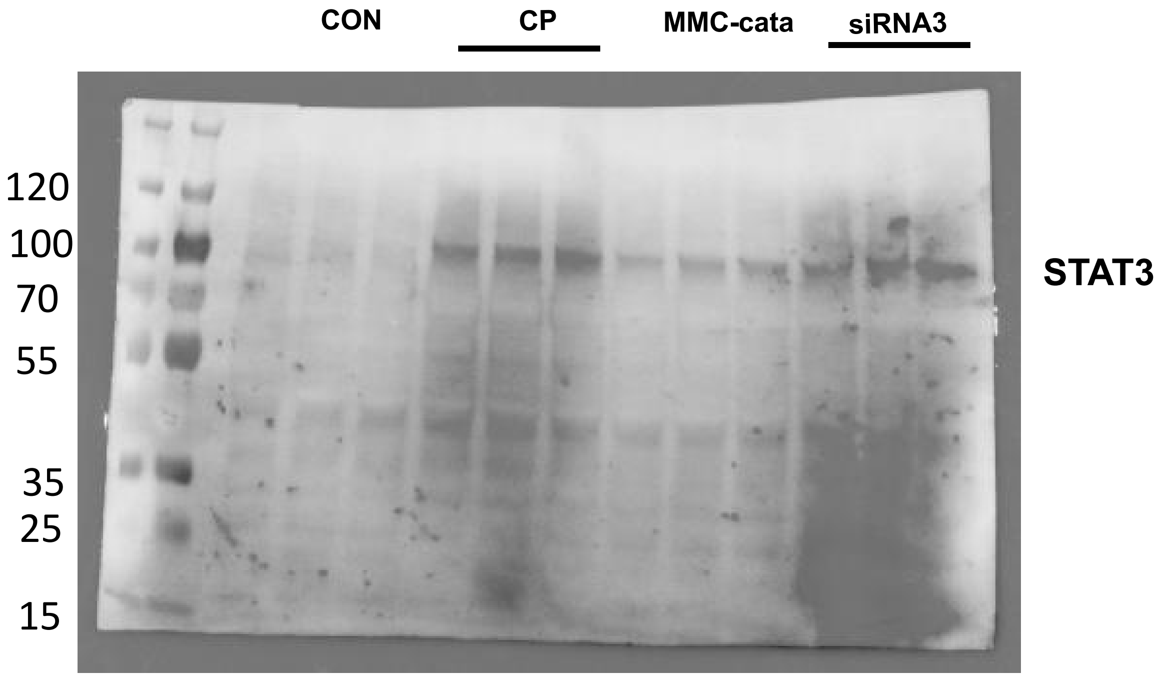

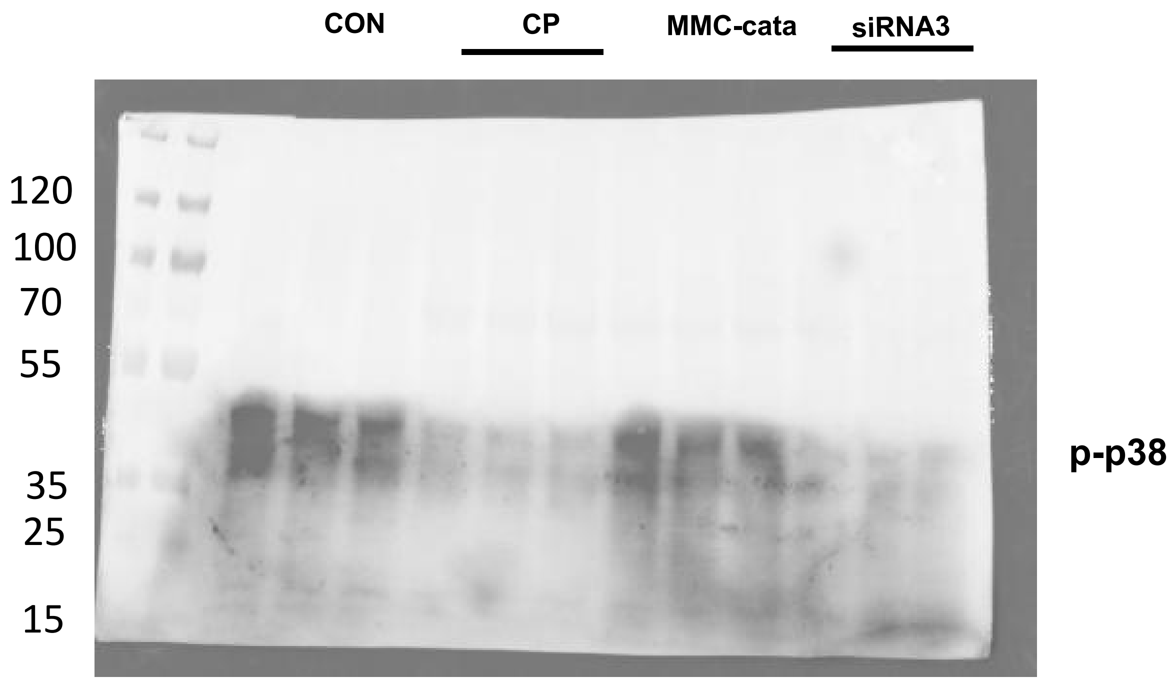

Uncropped gels for Western Blots in Figure 8

Figure 8A

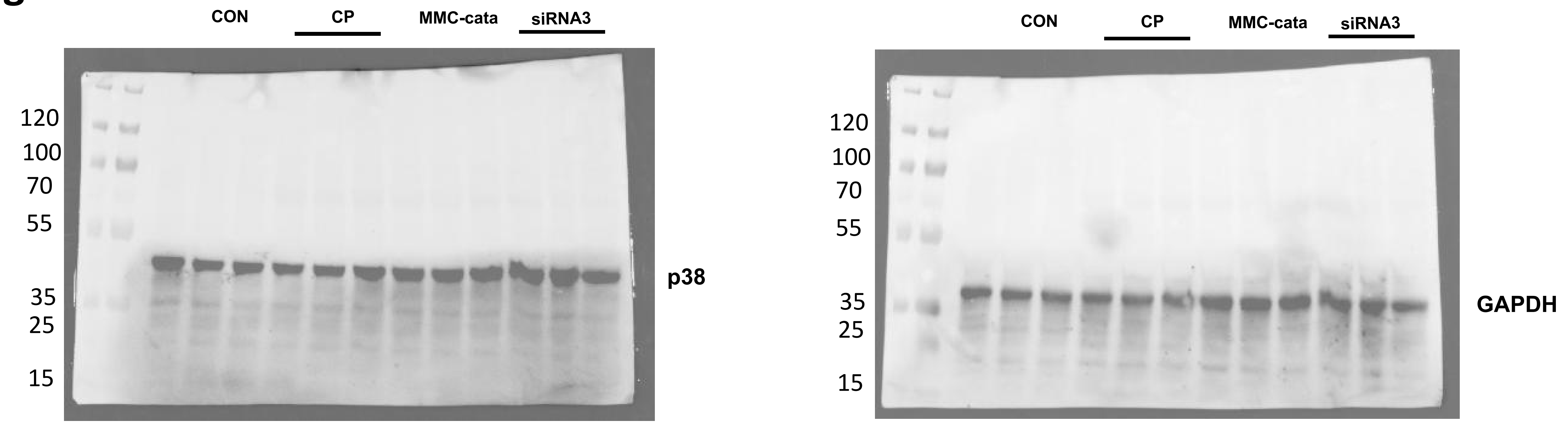

Figure 8C

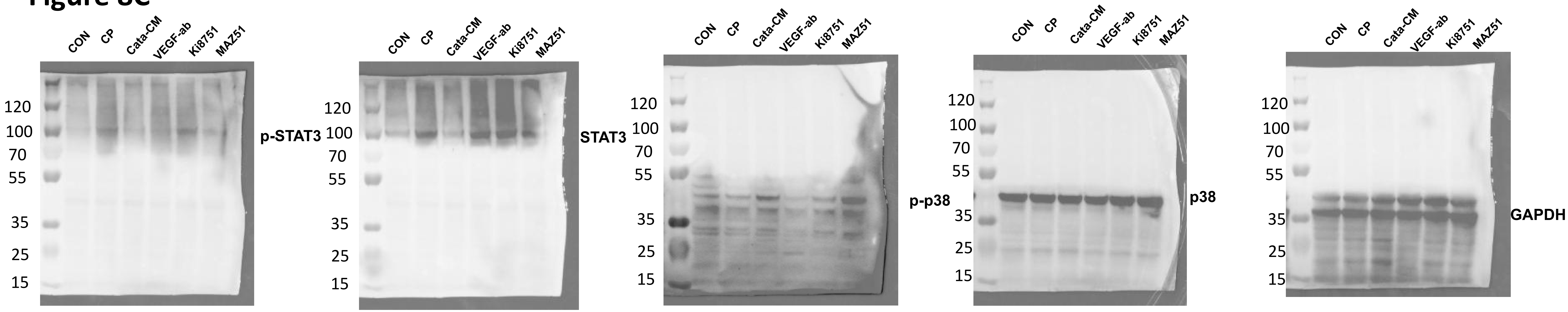

Uncropped gels for Western Blots in Figure 8

Figure 8E

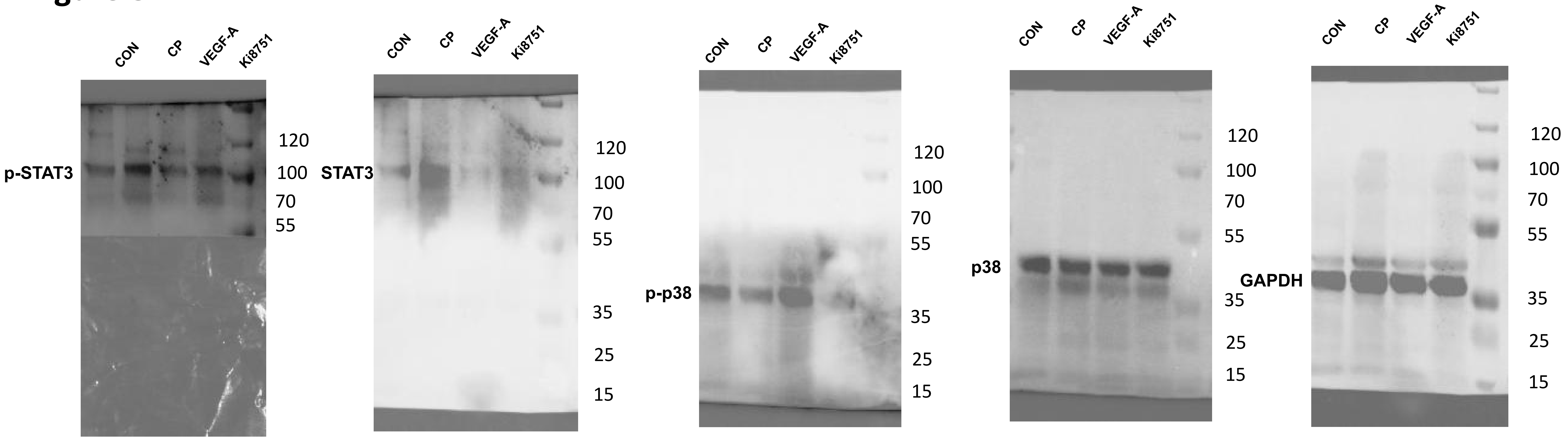

Uncropped gels for Western Blots in Figure 9

Figure 9K

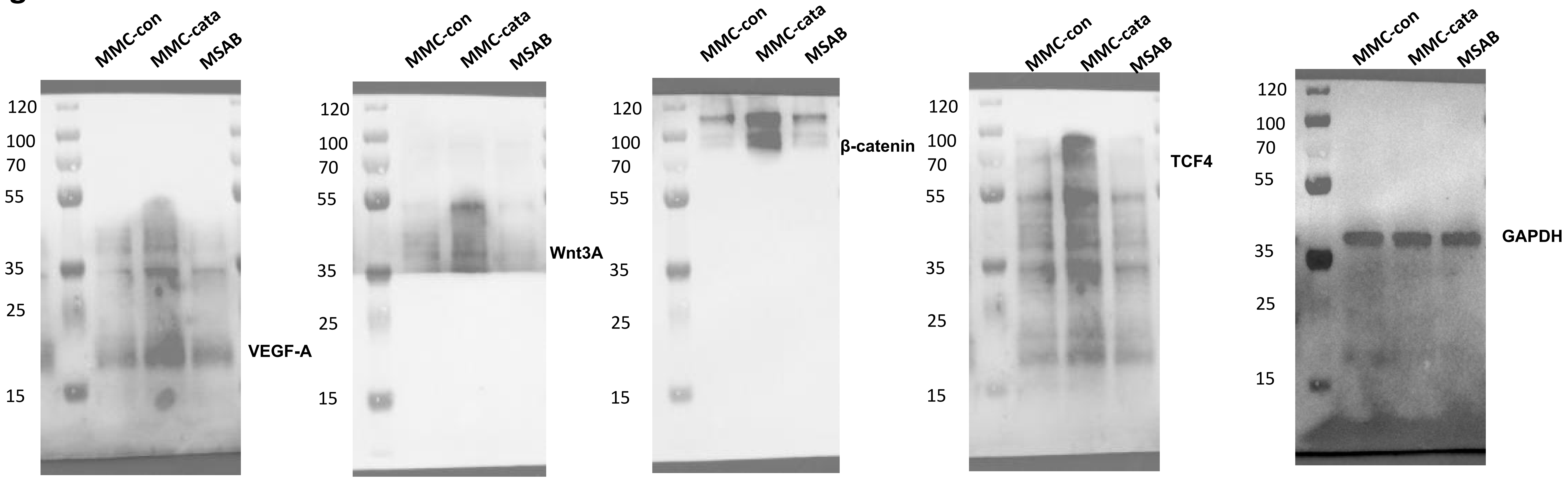

Supplement: Supplementary file 1 — Supplementary Material 1. [file 13287_2026_4914_MOESM1_ESM.pdf]
