# Supplementary material for: Pretreatment of metanephric mesenchymal cells with catalpol mitigates acute kidney injury through VEGF-A secretion via multiple mechanisms
Source: Stem Cell Res Ther. 2026 Mar 28;17:175. doi: 10.1186/s13287-026-04914-9 (PMC13151338; doi:10.1186/s13287-026-04914-9)
Supplement: Supplementary file 2 — Supplementary Material 2. [file 13287_2026_4914_MOESM2_ESM.docx]

**Supplementary Figures**


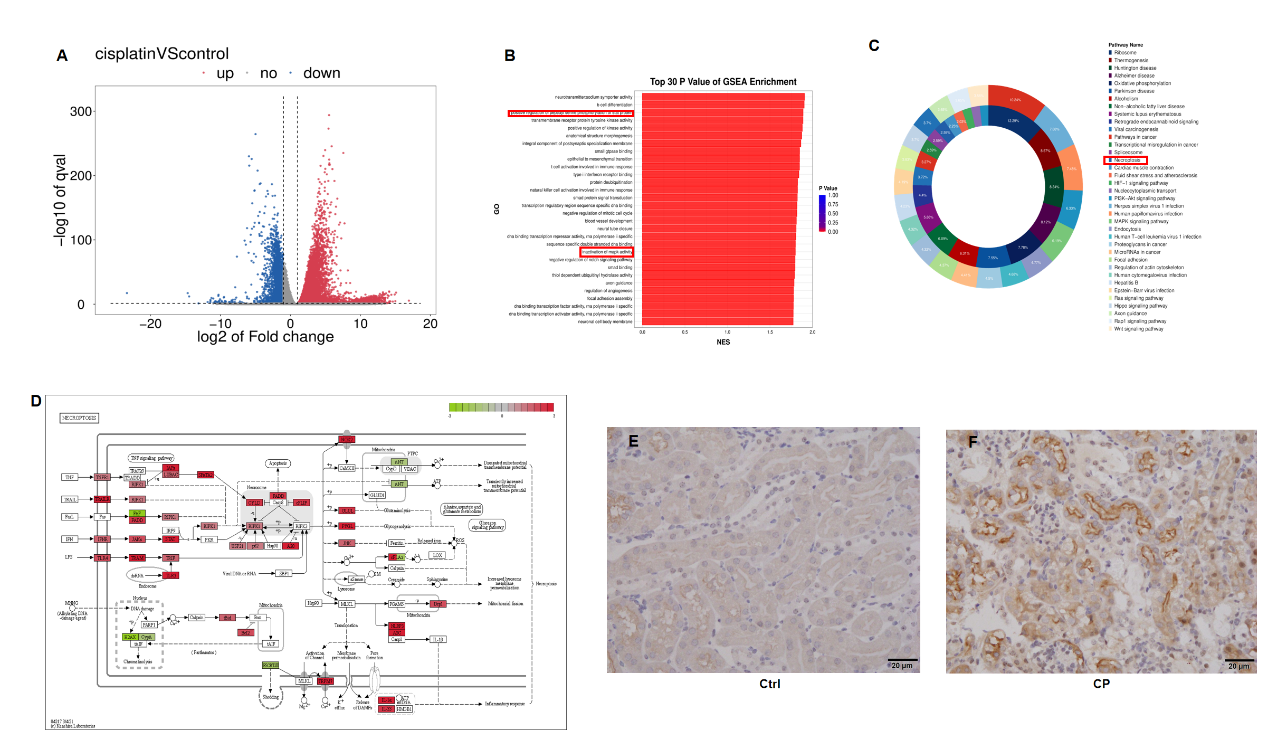
 **Supplementary Figure S1. Cisplatin induces necroptosis in renal tubular epithelial cells, causing damage through multiple pathways.** (A) RNA-seq volcano plot shows differentially expressed genes in cisplatin-treated vs. normal TCMK-1 cells (n=3). Red: upregulated; blue: downregulated; gray: no change. (B) GSEA of GO analysis reveals Stat protein phosphorylation pathway activation and MAPK pathway inhibition. (C-D) KEGG analysis shows necroptosis pathway activation and increased necroptosis-related gene expression in cisplatin-treated cells. (E-F) Immunohistochemistry shows MLKL expression at the damaged tubular epithelial cells (400x).


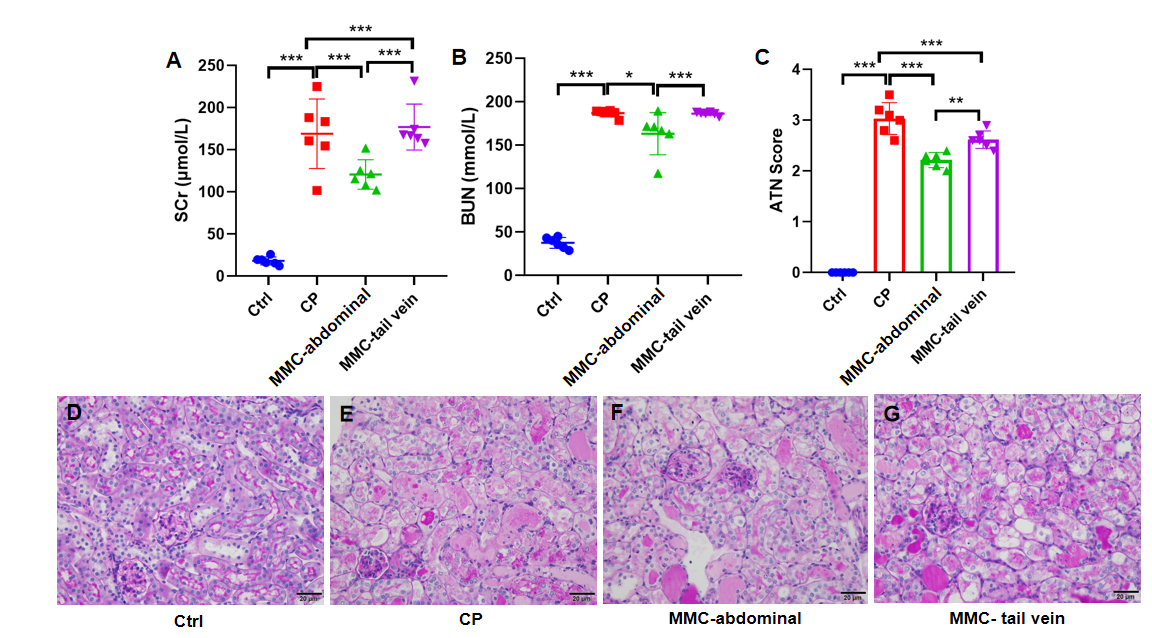
**Supplementary Figure S2. Intraperitoneal MMC injection is more effective than tail vein injection in alleviating cisplatin-induced acute kidney injury.** (A–B) Serum creatinine (SCr) and blood urea nitrogen (BUN) levels (n=6). (C–G) Kidney tissue sections stained with PAS (400x) and semi-quantitative histological scoring (n=6). Ctrl: control group; CP: cisplatin model group; MMC-abdominal: intraperitoneal MMC treatment group; MMC-tail vein: tail vein MMC treatment group. ***P < 0.001, **P < 0.01, *P < 0.05.

**
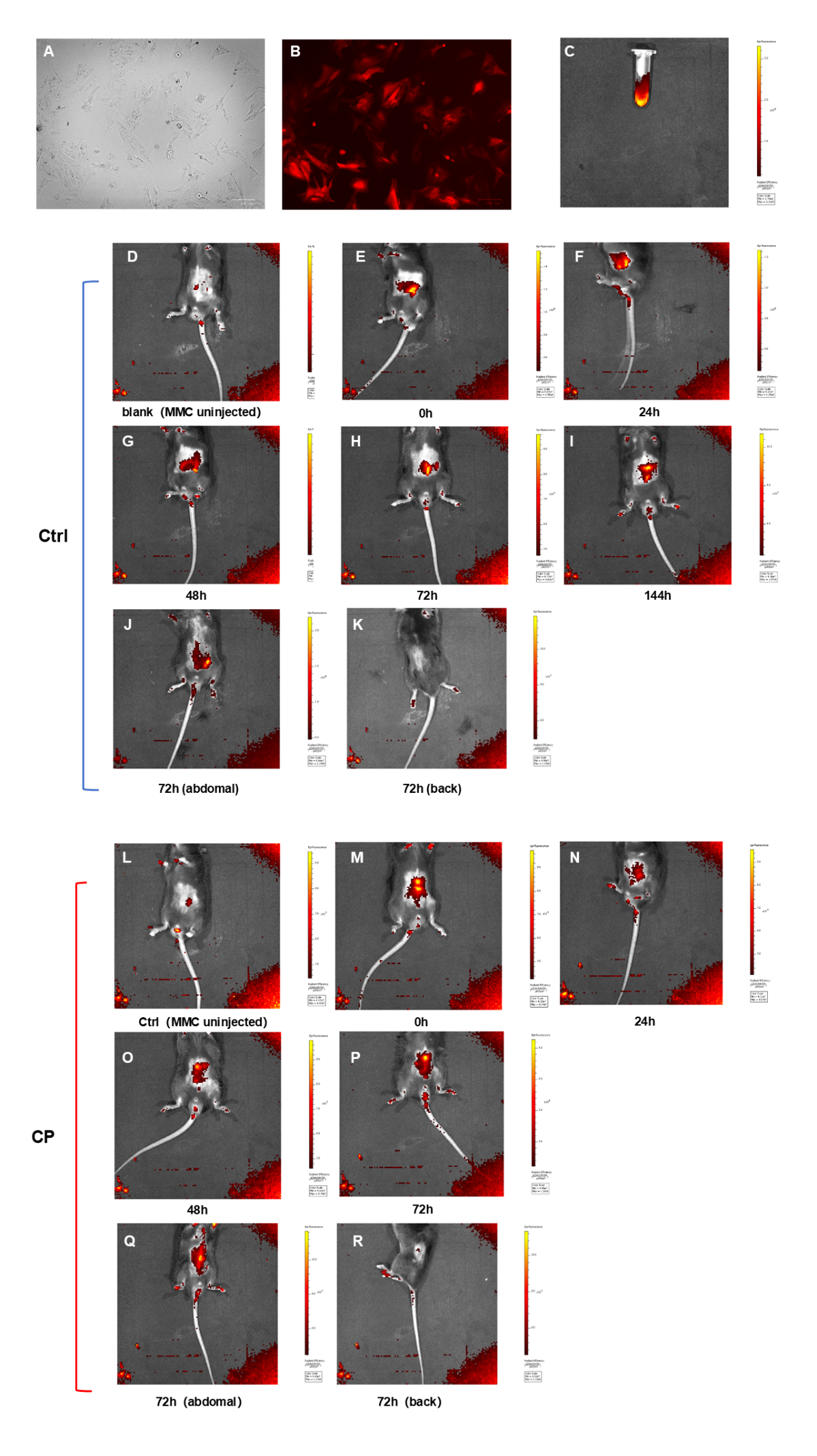
**

**Supplementary Figure S3**. **Catalpol-pretreated MMC remains primarily at the injection site after intraperitoneal administration.** (A-B) MMC-mCherry images under light and fluorescence microscopy (400x). (C) MMC-mCherry shows strong signals under small animal in vivo imaging. (D-K) In wild-type mice without cisplatin, MMC-mCherry remains at the injection site for 7 days without migrating to the kidneys, as observed by in vivo imaging. (L-R) In cisplatin-treated mice, MMC-mCherry also remains at the injection site without kidney migration, as observed by in vivo imaging.

**Supplementary Tables**

**Supplementary Table S1. Interaction information between Wnt3A and catalpol**

| **Amino Acid Site** | **Force Category** | **Distance** |
| --- | --- | --- |
| **ILE97** | Hydrophobic | 3.61 |
| **PRO46** | Hydrogen | 2.18 |
| **GLY47** | Hydrogen | 2.33 |
| **GLN52** | Hydrogen | 2.62 |
| **HIS113** | Hydrogen | 3.19 |
| **SER117** | Hydrogen | 3.19 |
| **ALA170** | Hydrogen | 2 |
| **ASN188** | Hydrogen | 2.6 |
| **ARG173** | Hydrogen | 2.44 |
|  | Salt Bridges | 4.12 |

| **Gene** | **Forward Primer Sequences** | **Reverse Primer Sequences** |
| --- | --- | --- |
| **VEGF-A sus 470 (siRNA3)** | GAUCCGCAGACGUGUAAAUTT | AUUUACACGUCUGCGGAUCTT |
| **VEGF-A sus 371 (siRNA2)** | CAGCACAACAAAUGUGAAUTT | AUUCACAUUUGUUGUGCUGTT |
| **VEGF-A sus 198 (siRNA1)** | CCGAUGAGAUCGAGUACAUTT | AUGUACUCGAUCUCAUCGGTT |
| **Negative control（NC）** | UUCUCCGAACGUGUCACGUTT | ACGUGACACGUUCGGAGAATT |

**Supplementary Table S2. Sequences of siRNAs.**
